# Efficacy and safety of tiotropium in partial and uncontrolled preschool asthma (TIPP): a phase III, multicentre, randomised, double-blind, placebo-controlled trial

**DOI:** 10.1016/j.eclinm.2026.104172

**Published:** 2026-09-04

**Authors:** Stefan Zielen, Nadine Wollscheid, Tanja Nickolay, Caroline Grimmel, David Scheele, Franziska Sattler, Freerk Prenzel, Michael Lorenz, Bianca Schaub, Christiane Lex, Franziska Stieglitz, Jordis Trischler, Helena Donath, Susanne Lau, Eckard Hamelmann, Christian Vogelberg, Michael Gerstlauer, Ruth Grychtol, Ralf Schubert, Monika Gappa, Lukas Schollenberger, Julia Wosniok

**Affiliations:** a Department for Children and Adolescents, Goethe-University Frankfurt, Frankfurt, Germany; b Institute of Respiratory Research, Medaimun GmbH, Frankfurt 60596, Germany; c Interdisciplinary Centre for Clinical Trials (IZKS), University Medical Centre Mainz, Mainz, Germany; d Children's Hospital, Evangelisches Krankenhaus Düsseldorf, Düsseldorf, Germany; e Paediatric Allergology, Department of Paediatrics, Dr. von Hauner Children’s University Hospital, LMU Munich-Member of the German Centre for Lung Research-DZL-LMU Munich, Munich, Germany; f Department of Paediatrics, Medical Centre, University of Leipzig, Leipzig, Germany; g Department of Paediatrics, Jena University Hospital, Friedrich-Schiller-University, Jena, Germany; h Division of Paediatric Cardiology and Intensive Care, Department of Paediatrics and Adolescent Medicine, University Medicine Göttingen, Göttingen, Germany; i Kinderlungen-Facharzt, Gemeinschaftspraxis in Mannheim, Mannheim, Germany; j Department of Respiratory Medicine, Immunology and Critical Care Medicine, Charité Universitätsmedizin Berlin, Berlin, Germany; k Department of Paediatrics, Children’s Centre Bethel, University Medicine, OWL, University Bielefeld, Bielefeld, Germany; l Department of Paediatrics, Faculty of Medicine and University Hospital Carl Gustav Carus, Technische Universität Dresden, Dresden, Germany; m Kinderklinik, Universitätsklinikum Augsburg, Germany; n Department of Paediatric Pneumology, Allergology, Neonatology, Hannover Medical School, Hannover, Germany; o Biomedical Research in Endstage and Obstructive Lung Disease (BREATH), Member of the German Centre for Lung Research (DZL), Hannover, Germany

**Keywords:** Preschool asthma, Uncontrolled asthma, Placebo-controlled trial, Paediatric care giver diary (PACD), Tiotropium bromide, Inhaled corticosteroids (ICS)

## Abstract

**Background:**

Preschool children with asthma are more susceptible to develop acute life-threatening respiratory distress leading to hospital admissions compared to school children and treatment options are limited for this age group. We evaluated the safety and efficacy of tiotropium as add-on therapy to inhaled corticosteroids (ICS) in preschool children with high-risk, partly controlled or uncontrolled asthma.

**Methods:**

TIPP was a phase III, prospective, multicentre, randomised, double-blind, placebo-controlled, parallel-group (investigator-initiated) trial conducted at 13 German centres (12 hospitals, one specialised medical practice). Children aged 1–5 years with physician-diagnosed asthma, partly controlled or uncontrolled symptoms despite ICS, and a history of severe exacerbations were randomly assigned to receive once-daily tiotropium (two puffs of 1.25 μg) via Respimat® inhaler or placebo as add-on to ICS therapy over 52 weeks. The primary outcome was time to first severe asthma exacerbation requiring hospitalisation and/or systemic corticosteroid treatment. All randomised participants were included in the intention-to-treat analysis and analysed by treatment received for safety. No primary or safety data were missing; therefore, no imputation was required. The study was registered in the “EU Clinical Trials Registry” (EudraCT 2021-000190-81).

**Findings:**

Between February 2022 and March 2025, 100 of the planned 204 children were enrolled, of whom 86 were randomised to tiotropium + ICS (n = 44) or placebo + ICS (n = 42). Mean age was 3.3 years (Standard deviation (SD) 1.3). The primary outcome time to first severe asthma exacerbation did not differ between groups (Hazard ratio (HR): 0.99, 95% confidence interval (CI): 0.51–1.92; p = 0.98). At least one adverse event was reported in 41 (93%) of 44 children in the tiotropium + ICS group and 42 (100%) of 42 children in the placebo + ICS group. 397 adverse events were reported with tiotropium + ICS group and 450 with placebo + ICS group. Tiotropium was well tolerated, and no new safety signal was identified.

**Interpretation:**

Tiotropium did not reduce the risk of a severe asthma exacerbation, but was well tolerated as add-on therapy to ICS in preschool children with high-risk asthma.

**Funding:**

German Federal Ministry of Education and Research (01KG2030); study medication provided by Boehringer Ingelheim Pharma GmbH & Co. KG (0205-0546).

**Research in context**

**Evidence before this study**

There is a high unmet need for better long-term treatment in preschool children with asthma, an age group which experiences a disproportionately high burden of severe asthma exacerbations leading to emergency department visits and hospital admissions compared with older children.

We searched PubMed for English-language publications from January 1, 1990, to December 31, 2021, using combinations of the terms “tiotropium bromide”, “asthma”, “wheeze”, “children”, “preschool”, “infant”, “toddler”, “adolescent”, and “Respimat”. The search yielded 27 records; however, only one prospective trial and two retrospective observational studies specifically investigated tiotropium in preschool children with asthma. Evidence for add-on treatment is limited with only three recent studies using tiotropium in preschool children. In these studies, tiotropium added to inhaled corticosteroids (ICS), with and without additional controller medication, was well tolerated and showed potential to reduce the risk of asthma exacerbations compared to placebo. However, the findings of these studies were limited due to the small sample size, duration and descriptive statistical analyses.

**Added value of this study**

This investigator-initiated, randomised, placebo-controlled trial evaluated tiotropium as add-on therapy to ICS over 52 weeks in preschool children with high-risk asthma. Tiotropium did not reduce the risk of a severe asthma exacerbation. The secondary and post-hoc analyses did not provide evidence of efficacy. Adequately powered studies are required to determine whether tiotropium has a clinically meaningful effect in this population. Importantly, no new safety signals were identified, supporting the known safety profile of tiotropium in this age group.

**Implications of all the available evidence**

The current evidence is insufficient to support routine use of tiotropium as an adjunct to ICS in preschool children with partially controlled or uncontrolled asthma. Tiotropium was well tolerated but efficacy was not demonstrated for the primary endpoint time to first severe exarcerbation. Adequately powered trials are required before any conclusions regarding efficacy or clinical recommendations can be drawn.

## Introduction

Recurrent obstructive bronchitis and asthma are among the most common chronic conditions in preschool children, and are major reasons for day care absenteeism, emergency department visits, and hospitalisations.^1^, ^2^, ^3^ Inhaled short-acting beta-2 agonists and inhaled corticosteroids (ICS) are the cornerstones of asthma treatment in children aged 1–5 years.^4^, ^5^, ^6^ However, a substantial proportion of children in this age group cannot be adequately controlled with ICS alone, which poses a considerable challenge for healthcare systems.^7^ Poor asthma control impairs patients' quality of life and increases the risk of future exacerbations, leading to greater healthcare utilisation and higher costs.^8^^,^^9^ In addition, young children are more susceptible to severe, sometimes life-threatening respiratory distress during asthma exacerbations compared to older children due to their smaller airways and potentially increased bronchial reactivity.^10^, ^11^, ^12^ This explains the relatively high rates of emergency department visits and hospital admissions in preschool-aged children with obstructive airway diseases.^13^^,^^14^ Treatment with ICS is less effective in preschool children, and the addition of long-acting beta agonists (LABAs) to ICS has not been shown to significantly reduce exacerbations requiring systemic steroid treatment.^15^, ^16^, ^17^ Furthermore, optimising asthma treatment in preschool children by increasing the ICS dose at the first signs of deterioration does not reduce the rate of severe asthma exacerbations, but bears the risk to reduce height growth.^18^^,^^19^ Thus, there is an unmet need to optimise asthma treatment in this vulnerable age group.

Tiotropium, a long-acting muscarinic receptor antagonist (LAMA), induces bronchodilation and reduces mucus secretion.^20^ Tiotropium is well established for the treatment of asthma and acts as an adjunct therapy to ICS in patients aged ≥ 6 years with severe asthma who have experienced one or more severe asthma exacerbations in the past 12 months.^21^^,^^22^ Its 24-h efficacy profile with a strong bronchodilatatory effect may be particularly beneficial for young children who experience acute nocturnal symptoms of airway obstruction, in the context of uncontrolled moderate to severe asthma. Currently, limited data are available to recommend tiotropium as an add-on treatment in preschool children with partially controlled or uncontrolled asthma, which is insufficient to justify its inclusion in current guidelines and disease management strategies for this age group.^23^, ^24^, ^25^

## Methods

### Study design and participants

In this prospective randomised, double-blind, placebo-controlled, parallel-group, phase III, investigator-initiated trial, we evaluated the safety and efficacy of tiotropium inhalation (2 puffs of 1.25 μg = 2.5 μg total daily dose) as an add-on to ICS in children aged 1–5 years with severe preschool asthma over a 52-week period.

The 2.5 μg once-daily dose was selected based on the available preschool paediatric tiotropium data, including the trial by Vrijlandt et al., in which 2.5 μg and 5 μg were evaluated in children aged 1–5 years, and both doses showed acceptable tolerability. Given the young age of the study population, and the long treatment duration, the lower dose was chosen for long-term add-on evaluation. The trial duration was expected to be 36 months with 24 months for recruitment, and 12 months for the treatment phase. Recruitment started in February 2022, and the trial ended in March 2025. We recruited 100 children at 13 centres in Germany (12 hospitals, one specialised medical practice). Children were eligible for inclusion if, in the 24 months prior to study entry, they had experienced at least one severe exacerbation requiring hospitalisation and/or at least two courses of systemic corticosteroid therapy. Intake of additional controller medication as LABAs or leukotriene receptor antagonists (LTRAs) was permitted. Patients were eligible if they had physician-diagnosed asthma for at least six months, defined by a history of asthma symptoms, including (but not limited to) wheezing, cough, and/or shortness of breath. In addition, all patients were required to have partially controlled or uncontrolled asthma, as defined by the Global Initiative for Asthma (GINA) guidelines, prior to screening (visit 1) and randomisation (visit 2), despite receiving ICS treatment for at least 4 weeks.^6^ In addition, background maintenance therapy with ICS and any pre-existing controller medication should remain unchanged throughout the study in accordance with the protocol, unless a temporary adjustment was clinically necessary during an exacerbation or for safety reasons.

Exclusion criteria included chronic diseases other than asthma, such as (but not limited to) cystic fibrosis, bronchopulmonary dysplasia, primary immunodeficiency, and congenital heart disease; clinically relevant abnormal screening haematology or blood chemistry; known hypersensitivity to anticholinergic drugs, or any component of the tiotropium inhalation solution; and severe acute asthma exacerbation, defined as systemic steroid intake or hospitalisation in the four weeks before visit 1. All inclusion and exclusion criteria are described in detail in the Supplement.

The study was registered in the EU Clinical Trials Registry (EudraCT 2021-000190-81, https://www.clinicaltrialsregister.eu) and transitioned in accordance with EU Regulation 536/2014 on clinical trials (EU CT 2024-513916-84-00). The complete study protocol is provided in the Supplementary Material.

### Ethics statement

The study was conducted in accordance with the principles of the Declaration of Helsinki, the International Council for Harmonisation Good Clinical Practice guidelines (ICH-GCP), and all applicable local regulatory requirements. The Ethics Committee of the Goethe-University Frankfurt (application number 2021-443-AMG), and all participating Ethics Committees, approved the study. An independent Data Monitoring Committee (DMC) was established and operated according to a prespecified DMC charter. The DMC reviewed accumulating safety data and overall trial conduct to ensure patient safety, data integrity, and ethical conduct. Written informed consent was obtained from both parents, or the custodial parent, before the child's participation.

### Randomisation and masking

A randomisation list with a 1:1 allocation ratio was generated by an independent person at the coordinating centre, the Interdisciplinary Centre for Clinical Trials (IZKS) Mainz, using a SAS®-based computer program with permuted blocks, stratified by study site. Block sizes were variably sized (2 and 6) and concealed.

The randomisation list was kept in safe and confidential custody at IZKS Mainz. The study was conducted in a double-blind manner; all participants, study personnel and data analysts were blinded to treatment allocation. Tiotropium and placebo solution were provided by Boehringer Ingelheim and administered by identical Respimat® inhalers using a spacer with mask or mouth piece as appropriate, ensuring that patients, caregivers, investigators, and all other study personnel remained blinded to treatment assignment.

### Procedures

Patients who met all inclusion criteria and none of the exclusion criteria were randomised at visit 2 into the 52-week treatment period and assigned randomly to one of two treatment groups (tiotropium + ICS vs. placebo + ICS). Additional clinic visits were scheduled at 6, 16, 28, 40, and 52 weeks after the start of therapy. Telephone contacts were scheduled between visits 4 and 5, 5 and 7, and 6 and 7.

At each visit, a physical examination, including measurements of height, weight, and vital signs, was performed. The paediatric caregiver diary (PACD) was used to assess daily asthma symptoms.^26^ The PACD responses were recorded electronically using a custom-developed diary app (https://play.google.com/store/apps/details?id=com.kgu.tippdiary&hl). The diary comprises three questions to be answered each morning upon waking, and seven questions to be answered each evening immediately after the child goes to bed. The combined daily score is the average of the scores from questions 4–7 in the diary, which assess the severity of coughing, wheezing, breathing difficulties, and activity limitations. All questions were scored on a scale from 0 (best) to 5 (worst). The test for respiratory and asthma control in Kids (TRACK) was used to evaluate respiratory and asthma control at visit 1, visit 2 and visit 4, and at the end of study at visit 7.^27^ Caregivers evaluated the overall treatment effect using the Caregiver Global Impression of Change (CGI-C) at the end of the 52-week treatment period.^28^

Pulmonary function tests (PFTs) were performed only in children aged ≥ 4 years who were able to perform forced expiratory and tidal breathing manoeuvres of acceptable quality. Spirometry was conducted according to the American Thoracic Society/the European Respiratory Society (ATS/ERS) guidelines.^29^ All PFTs were repeated until three technically acceptable measurements were obtained, and the best of these three measurements was recorded. The following variables were documented: Vital capacity (VC), forced expiratory volume in 1 s (FEV_1_), forced vital capacity (FVC), and FEV_1_/FVC (Tiffenau-Index), each expressed in litres and as percentage of predicted [L, % pred]. Calibration of PFT equipment was mandatory on each visit day.

Safety laboratory parameters, including complete blood count with eosinophils, total IgE, and specific IgE to ten allergens (birch, grass, house dust mites (*Dermatophagoides pteronyssinus* and *farinae*), *Alternaria*, *Cladosporium*, cat, dog, milk, egg, and peanut) were collected at the beginning (visit 1) and end of the trial (visit 7).

### Outcomes

The objective of this study was to evaluate whether the addition of tiotropium to ICS can prevent severe exacerbations and reduces healthcare utilisation (hospitalisations, physician visits, and antibiotic use) in partially controlled and uncontrolled preschool asthma. The primary efficacy endpoint was time to first severe exacerbation requiring hospitalisation and/or at least three days of treatment with systemic steroids, or one day of rectal applied prednisolone (Rectodelt®).

Secondary outcomes for all patients included: number of severe exacerbations; severe exacerbation-free survival time; percentage of patients requiring systemic corticosteroid rescue for asthma during the 52-week treatment period; number of hospitalisations; percentage of days and episodes of ≥ 3 days with salbutamol rescue use (mild exacerbations); percentage of days without asthma symptoms assessed by the TIPP diary app, percentage of night-time awakenings, number of physician visits during treatment, number of missed days in daycare, number of asthma-related events defined by the use of antibiotics, evaluation of the TRACK, CGI-C at the end of treatment. Additionally, two post-hoc analyses were performed on number of exacerbations and exacerbation-free survival (both mild and severe exacerbations combined). The following secondary endpoints were assessed for children aged ≥ 4 years only: Lung function outcomes included post-dose FEV_1_ and FVC (30 min after investigational medicinal product (IMP) use) relative to pre-dose baseline (visit 2), as well as trough FEV_1_ and FVC (defined as measurements obtained ∼24 h after the last IMP dose). A response was defined as a ≥ 10% improvement from baseline. Potential biomarkers for treatment response included eosinophil counts, total IgE levels, and allergen-specific IgE (sIgE) to common aero- and food allergens.

Safety was assessed by evaluating symptom scores, rescue medication use recorded in the electronic diary, and the incidence of adverse events (AEs).

### Statistical analysis

The primary endpoint—time to first severe asthma exacerbation—was analysed using a Cox proportional hazards model, adjusted for treatment group, age group (1–3 vs. 4–5 years), and study centres (Frankfurt vs. other centres). Results were presented as hazard ratios (HR) with 95% confidence intervals (CIs). Sensitivity analyses additionally evaluated recurrent severe exacerbations using a Prentice–Williams–Peterson model. Secondary endpoints and post-hoc analyses (e.g., number of exacerbations, hospitalisations, asthma-related events) were analysed using negative binomial regression, adjusting for treatment group, age group (1–3 vs. 4–5 years), and study centre (Frankfurt vs. other centres) with time in study included as an offset. Further categorical variables were evaluated by Fisher’s exact test for 2 × 2 contingency tables. Two-sided p-values were calculated as twice the exact one-sided probability based on the hypergeometric distribution. Continuous variables were evaluated using the t-test or Mann-Whitney-U-test, depending on their distribution. Subgroup analyses were performed for sex (male/female) and allergy status (allergic/non-allergic, based on specific IgE ≥ 0.75 kU/L). The Cox model of the primary analysis was extended accordingly, including treatment-by-subgroup interaction terms to assess effect modification.

**Fig. 1:**
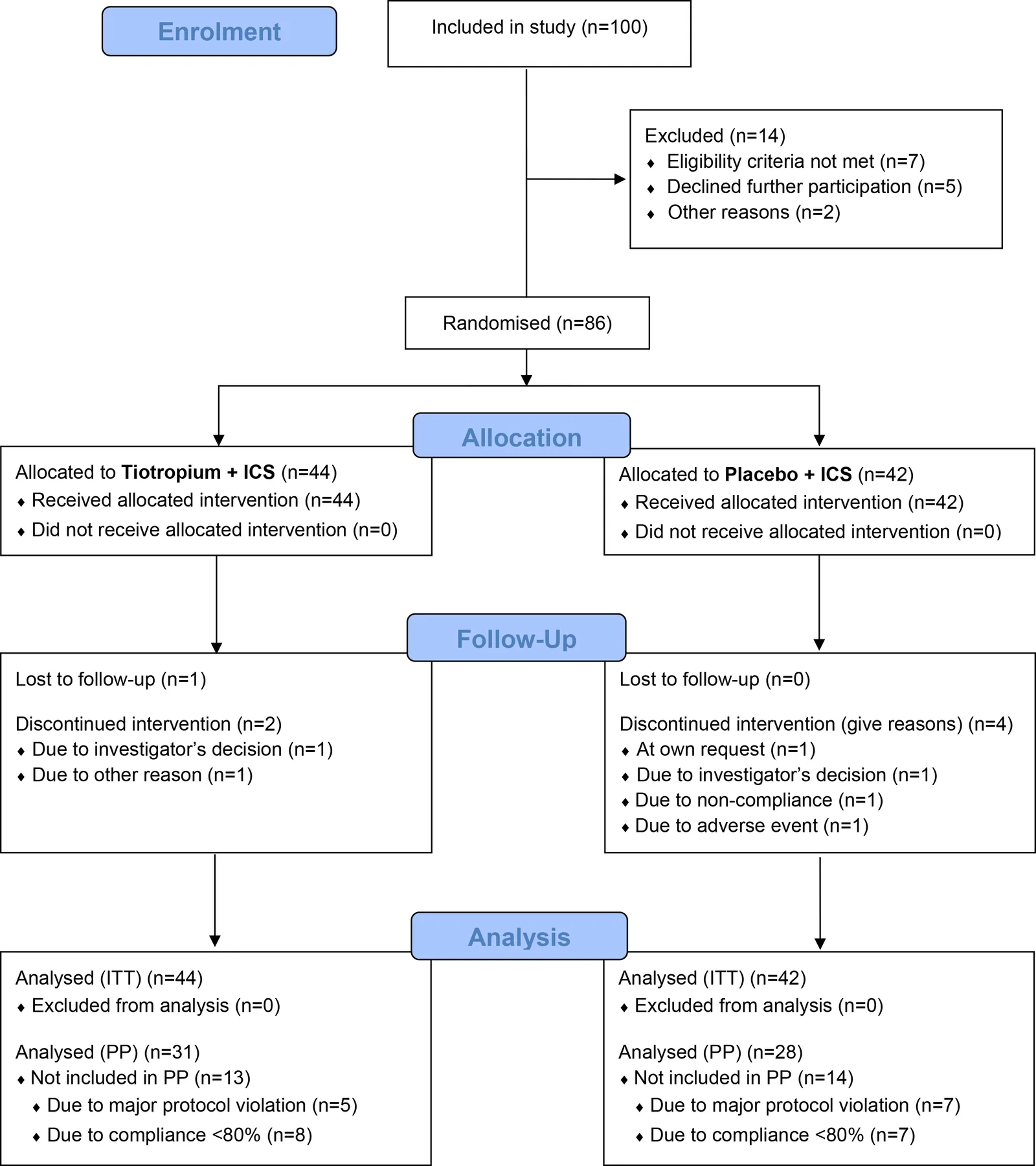
Study flow chart.

Kaplan–Meier curves were generated for time-to-event outcomes. All randomised participants were included in the intention-to-treat (ITT) population, which was used for all primary and secondary analyses covering the first 365 days after treatment initiation, while safety analyses were conducted according to treatment received. No primary or safety data were missing; therefore, no imputation was required. The per-protocol (PP) population was defined as a supportive analysis population and was not used for the primary efficacy analysis. Although the PP population was predefined, no separate efficacy analyses in this population are presented because the study was substantially underpowered following reduced recruitment, and the ITT population remained the prespecified primary analysis population. For completeness the characteristics of the PP population are shown in Fig. 1 and Supplemental Tables S1 and S2.

The study centre was included as a covariate in all major analyses to statistically control for centre-related differences. However, due to the small number of patients per centre, no formal effect analysis was performed. No correction for multiple testing was applied. All tests were two-sided with a significance level of α = 0.05. Secondary endpoints and post-hoc analyses were considered in support of the primary analysis; the p-values were interpreted exploratorily. No interim analyses were performed. All analyses were performed using SAS® version 9.4.

For the study sample size, it was assumed that most patients treated with ICS + placebo suffered from one severe exacerbation within one year, therefore a rate of 90% of placebo patients with a severe exacerbation after one year was anticipated. For the tiotropium group, a rate of 75% was assumed. The estimation of the difference in rates between the placebo and the tiotropium group was performed conservatively, assuming a smaller difference than observed after 105 days in the study of Vrijlandt et al.^23^ The assumed dropout rate of 15% was converted into a group-loss hazard (0.16) and considered in the sample size calculation. With an event rate of 90% in the control group and 75% in the tiotropium group and a drop-out-rate of 15%, the sample size amounted to 152 patients when applying a two-sided log-rank test with a HR of 0.60, a significance level of alpha = 0.05 and a power of 0.8. The power calculation was performed with PROC POWER in SAS® 9.4.

Originally, it was planned to enrol 204 children (with a power of 90%) within one year. This target was not achieved due to delayed site initiations, reduced presentation of children with acute asthma symptoms during the COVID-19 pandemic, and increased incidence of influenza and respiratory syncytial virus (RSV) infections in fall 2022, which strained clinical capacity and limited staff availability. This impacted the overall study progress significantly. Consequently, the protocol was amended, the target sample size was reduced to 152 children, and the recruitment period was extended by one year. The study procedures and objectives remained unchanged. Due to slower-than-anticipated recruitment and a higher-than-expected rate of asthma exacerbations, the recruitment target was revised to 100 screened patients.

### Role of the funding source

The study was funded by a grant of the German Federal Ministry of Education and Research (01KG2030). The study was an independent, investigator-initiated study supported by Boehringer Ingelheim Pharma GmbH & Co. KG (0205-0546) providing tiotropium and placebo solution via Respimat® device. Boehringer Ingelheim had no role in study design, data collection, data analysis, data interpretation, or writing of the report.

## Results

### Demographics and baseline characteristics of the ITT population

Of the 100 enrolled patients, 86 were randomised to receive either tiotropium + ICS (n = 44) or placebo + ICS (n = 42) group; 14 patients were not randomised (Fig. 1, study flow chart). All randomised patients were included in the ITT and safety populations. The PP population comprised 31 patients (70% of the ITT population) in the tiotropium group, and 28 patients (67% of the ITT population) in the placebo group. The most common protocol deviations were treatment compliance <80% (17% [N = 15]), and the use of non-permitted medication (10% [N = 9]). Details are provided in the Supplement (Table 1, overview of TIPP study populations). The characteristics of the PP population were comparable to those of the ITT population, with no statistically significant differences in baseline characteristics. Details are shown in the Supplement (Table 2, age and sex distribution in the different populations).

**Table 1:** Demographics and baseline characteristics of the ITT population.

Variable	Value	Tiotropium + ICS (N = 44)	Placebo + ICS (N = 42)	Total (N = 86)
Age (years)	Mean (SD)	3.2 (1.3)	3.4 (1.3)	3.3 (1.3)
Sex	Male	23 (52%)	23 (55%)	46 (53%)
	Female	21 (48%)	19 (45%)	40 (47%)
Race	White	35 (80%)	34 (81%)	69 (80%)
	Asian	1 (2%)	0 (0%)	1 (1%)
	Black	0 (0%)	0 (0%)	0 (0%)
	Other	8 (18%)	8 (19%)	16 (19%)
BMI Z-Scores^b^	Mean (SD)	0.32 (0.84)	0.43 (1.04)	0.37 (0.94)
Gestational age (weeks)^c^	Mean (SD)	39.0 (1.8)	38.2 (3.0)	38.6 (2.4)
Breastfeeding (n)^c^	N (%)	39 (89%)	32 (78%)	71 (84%)
Allergic rhinitis (n)	N (%)	7 (16%)	9 (21%)	16 (19%)
Atopic dermatitis (n)	N (%)	3 (7%)	4 (10%)	7 (8%)
No Household pet	N (%)	31 (70%)	29 (69%)	60 (70%)
Dog	N (%)	4 (9%)	6 (14%)	10 (12%)
Cat	N (%)	8 (18%)	7 (17%)	15 (17%)
Guinea pig	N (%)	1 (2%)	0 (0%)	1 (1%)
Household/ second-hand smoke	N (%)	10 (23%)	11 (26%)	21 (24%)
History of severe exacerbations	> 12–24 months, Median (IQR)	1.0 (0.0–2.0)	1.0 (0.0–3.5)	1.0 (0.0–3.0)
	≤ 12 months, Median (IQR)	3.0 (2.0–5.0)	4.0 (2.0–6.0)	3.5 (2.0–6.0)
	Total numbers, Median (IQR)	4.0 (2.0–6.0)	5.5 (3.5–8.0)	4.5 (3.0–8.0)
Result of physical examination	Abnormal findings, NOT clinically significant	10 (23%)	6 (14%)	16 (19%)
	Abnormal findings, clinically significant^a^	7 (16%)	10 (24%)	17 (20%)
GINA: daytime symptoms	N (%)	38 (86%)	36 (86%)	74 (86%)
GINA: limitations of activities	N (%)	28 (64%)	29 (69%)	57 (66%)
GINA: nocturnal symptoms/awakening	N (%)	34 (77%)	33 (79%)	67 (78%)
GINA: need for reliever/ rescue treatment	N (%)	25 (57%)	25 (60%)	50 (58%)
Level of asthma symptom control	Well controlled	0 (0%)	0 (0%)	0 (0%)
	Partly controlled	18 (41%)	12 (29%)	30 (35%)
	Uncontrolled	26 (59%)	30 (71%)	56 (65%)
Time since asthma diagnosis (years)	Mean (SD)	1.3 (1.1)	1.3 (0.9)	1.3 (1.0)
ICS cumulative dose (μg)	Mean (SD)	166.2 (71.3)	223.8 (128.4)	194.7 (108.3)
ICS	N (%)	15 (34%)	11 (26%)	26 (30%)
ICS + LTRA	N (%)	1 (2%)	2 (5%)	3 (3%)
ICS + LABA	N (%)	23 (52%)	22 (52%)	45 (52%)
ICS + LABA + LTRA	N (%)	5 (11%)	7 (17%)	12 (14%)

*BMI*, Body mass index; *GINA*, Global Initiative for Asthma; *ICS*, Inhaled corticosteroids; *IQR*, Interquartile range; *ITT*, Intention-to-treat; *LABA*, Long-acting beta-2 agonists; *LTRA*, Leukotriene receptor antagonists; *N*, Number of patients; *SD*, Standard deviation.

Analysis Set: ITT Population (N = 86).

a Respiratory findings and eczema were recorded.

b BMI-Z-scores according to Kromeyer-Hausschild.^30^

c Missing values are not included in calculation of percentages. Missing data occurred for gestational age in the Tiotropium + ICS group (n = 1) and breastfeeding in the Placebo + ICS group (n = 1).

**Table 2:** Secondary efficacy outcomes and exploratory post-hoc analyses.

Outcome	Tiotropium + ICS	Placebo + ICS	Effect estimate (95% CI)	p-value
Exacerbation-related outcomes (Rate-based; negative binomial models)				
Severe exacerbations/patient-year (N = 86)	0.8 (35 events/42 PY)	1.1 (42 events/40 PY)	IRR 0.87 (0.44–1.73)	0.69
Mild exacerbations/patient-year (N = 86)	3.0 (126 events/42 PY)	3.9 (156 events/40 PY)	IRR 0.76 (0.54–1.07)	0.12
Hospitalisations/patient-year (N = 86)	0.3 (13 events/42 PY)	0.4 (16 events/40 PY)	IRR 0.82 (0.31–2.13)	0.68
Asthma-related events/ patient-year (N = 86)	1.3 (53 events/42 PY)	1.4 (55 events/40 PY)	IRR 0.89 (0.54–1.45)	0.65
Binary outcomes (Fisher’s exact test)				
Systemic corticosteroid use (N = 86)	21/44 (48%)	22/42 (52%)	–	0.83
FEV_1_ response ≥ 10% (N = 24)	11/12 (92%)	9/12 (75%)	–	0.59
FVC response ≥ 10% (N = 26)	9/13 (69%)	12/13 (92%)	–	0.32
Patient-reported outcomes (Non-parametric summaries; Wilcoxon tests)				
Salbutamol rescue use (% of days) (N = 86)	10.7 (3–18)	16.7 (7–29)	–	0.10
Symptom-free days (% of days) (N = 84)	67 (50–83)	61 (50–74)	–	0.27
Night-time awakenings (% of days) (N = 84)	5 (3–10)	9 (6–12)	–	0.024
Physician visits (N = 86)	0 (0–2)	0 (0–4)	–	0.078
Missed daycare days (N = 86)	17 (11–35)	27.5 (15–40)	–	0.32
Continuous outcomes (Two-sample t-test)				
TRACK score after 16 weeks (mean, SD) (N = 83)	71.6 (18.2)	63.1 (21.1)	–	0.054
TRACK score at end of study (mean, SD) (N = 83)	71.0 (27.5)	67.1 (28.3)	–	0.52
FEV_1_ change (L, mean, SD) (N = 20)	+0.008 (0.114)	−0.009 (0.127)	–	0.76
FVC change (L, mean, SD) (N = 23)	+0.035 (0.094)	−0.003 (0.132)	–	0.43
Exploratory post-hoc analyses (negative binomial models/ Cox regression)				
Any exacerbations/patient-year (N = 86)	3.4 (144 events/42 PY)	4.2 (167 events/40 PY)	IRR 0.82 (0.59–1.15)	0.25
Median time to first exacerbation (days) (N = 86)	61 (IQR 21–341)	35 (IQR 9–139)	HR 0.60 (0.37–0.98)	0.042

*CI*, Confidence interval; *FEV*_*1*_, Forced Expiratory Volume in 1 s; *FVC*, Forced Vital Capacity; *HR*, Hazard ratio; *ICS*, Inhaled corticosteroids; *IQR*, Interquartile range; *IRR*, Incidence rate ratio; *N*, Number of patients; *PY*, patient-year; *SD*, Standard deviation; *TRACK*, Test for Respiratory and Asthma Control in Kids.

Patient-reported outcomes are presented as median (IQR), whereas continuous outcomes are presented as mean (SD).

Mean age at randomisation was 3.3 (Standard deviation (SD) 1.3) years in both treatment groups. Sex distribution was balanced, with males comprising approximately 53% of the study population. Most children were white (80% [N = 69]), with smaller proportions identifying as Asian (1% [N = 1]) or other races (19% [N = 16]), with no meaningful differences between groups (Table 1). Anthropometric measurements, including weight and height, and mean gestational age at birth (38.6 weeks (SD 2.4)) did not differ between groups. A high proportion of children had been breastfed—89% (N = 39) in the tiotropium group, and 78% (N = 32) in the placebo group. Common comorbidities were allergic rhinitis (19% [N = 16]) and atopic dermatitis (8% [N = 7]), with comparable rates across treatment groups. Household pet exposure was similar, with 70% (N = 60) of children living in pet-free homes. Among those with pets, exposure included dogs (12% [N = 10]), cats (17% [N = 15]), and guinea pigs (1% [N = 1]).

Clinically significant abnormalities on physical examination, including respiratory findings and eczema, were observed in 20% (N = 17) of patients, occurring more frequently in the placebo + ICS group (24% [N = 10]) than in the tiotropium + ICS group (16% [N = 7]) (Table 1). Based on GINA criteria, 86% (N = 74) of all patients reported daytime symptoms, 66% (N = 57) reported limitations in daily activities, 78% (N = 67) reported nocturnal symptoms or awakening, and 58% (N = 50) required reliever or rescue medication. Overall, 35% (N = 30) of patients were classified as having partly controlled asthma, and 65% (N = 56) as having uncontrolled asthma.

Mean time since asthma diagnosis was 1.3 (SD 1.0) years in both groups. At baseline, 26 patients (30%) were receiving ICS monotherapy, three patients (3%) ICS + LTRA, 45 patients (52%) ICS + LABA, and 12 patients (14%) ICS + LABA + LTRA. Treatment patterns were similar across groups. In addition, as respiratory infections and exacerbations in preschool children are highly seasonal, we examined the seasonal distribution of referrals between the groups. No clinically significant imbalance in the referral season was observed between the treatment groups.

Baseline sensitisations were similar across treatment groups (Supplement, Table 3: Baseline characteristics of the ITT population—allergy diagnostics/total IgE, eosinophils, and sIgE). Median total IgE was 16 (Interquartile Range (IQR): 16–94) kU/L, with 23% (N = 18) of patients presenting elevated levels (≥ 100 kU/L). Median eosinophil counts were approximately 0.28 (IQR: 0.15–0.41) x10^3^/μL, and 44% (N = 23) of patients in each group had eosinophil levels > 300/μL. Sensitisation (sIgE ≥ 0.75 kU/L) rates ranged from 11% to 20% per allergen, with the highest rates observed for birch, house dust mite, and cat dander, although none exceeded 20% prevalence. Spirometry was not feasible in 53 patients. Overall, lung function was similar between groups.

**Table 3:** Adverse events in the treatment groups.

Parameter	Tiotropium + ICS (N = 44)		Placebo + ICS (N = 42)		Total (N = 86)	
	Patients with AE	Number of AEs	Patients with AE	Number of AEs	Patients with AE	Number of AEs
Any AE	41 (93%)	397 (100%)	42 (100%)	450 (100%)	83 (97%)	847 (100%)
Respiratory tract infection	8 (18%)	15 (3.8%)	11 (26%)	25 (5.6%)	19 (22%)	40 (4.7%)
Cough	8 (18%)	16 (4.0%)	6 (14%)	18 (4.0%)	14 (16%)	34 (4.0%)
Bronchitis	27 (61%)	98 (25%)	30 (71%)	122 (27%)	57 (66%)	220 (26%)
Pneumonia	8 (18%)	9 (2.7%)	12 (29%)	17 (3.8%)	20 (23%)	26 (3.1%)
Upper respiratory tract infection	1 (2.3%)	1 (0.3%)	8 (19%)	8 (1.8%)	9 (10%)	9 (1.1%)
Nasopharyngitis	24 (55%)	50 (13%)	15 (36%)	35 (7.8%)	39 (45%)	85 (10%)
Tonsillitis	2 (4.5%)	2 (0.5%)	5 (12%)	6 (1.3%)	7 (8.1%)	8 (0.9%)
Pyrexia	7 (16%)	13 (3.3%)	10 (24%)	16 (3.6%)	17 (20%)	29 (3.4%)
Dyspnoea	1 (2.3%)	1 (0.3%)	0 (0.0%)	0 (0.0%)	1 (1.2%)	1 (0.1%)
Headache	1 (2.3%)	1 (0.3%)	1 (2.4%)	1 (0.2%)	2 (2.3%)	2 (0.2%)
Diarrhoea	3 (6.8%)	4 (1.0%)	4 (9.5%)	4 (0.9%)	7 (8.1%)	8 (0.9%)
Vomiting	3 (6.8%)	3 (0.8%)	2 (4.8%)	2 (0.4%)	5 (5.8%)	5 (0.6%)
Insomnia	0 (0.0%)	0 (0.0%)	1 (2.4%)	1 (0.2%)	1 (1.2%)	1 (0.1%)

Patients may have experienced more than one event. Percentages for patients are based on the total number of patients per group, whereas percentages for events are based on the total number of adverse events.

*AE*, Adverse event; *ICS*, Inhaled corticosteroids; *N*, Number of patients.

Analysis set: ITT population (N = 86).

### Primary outcome

**Fig. 2:**
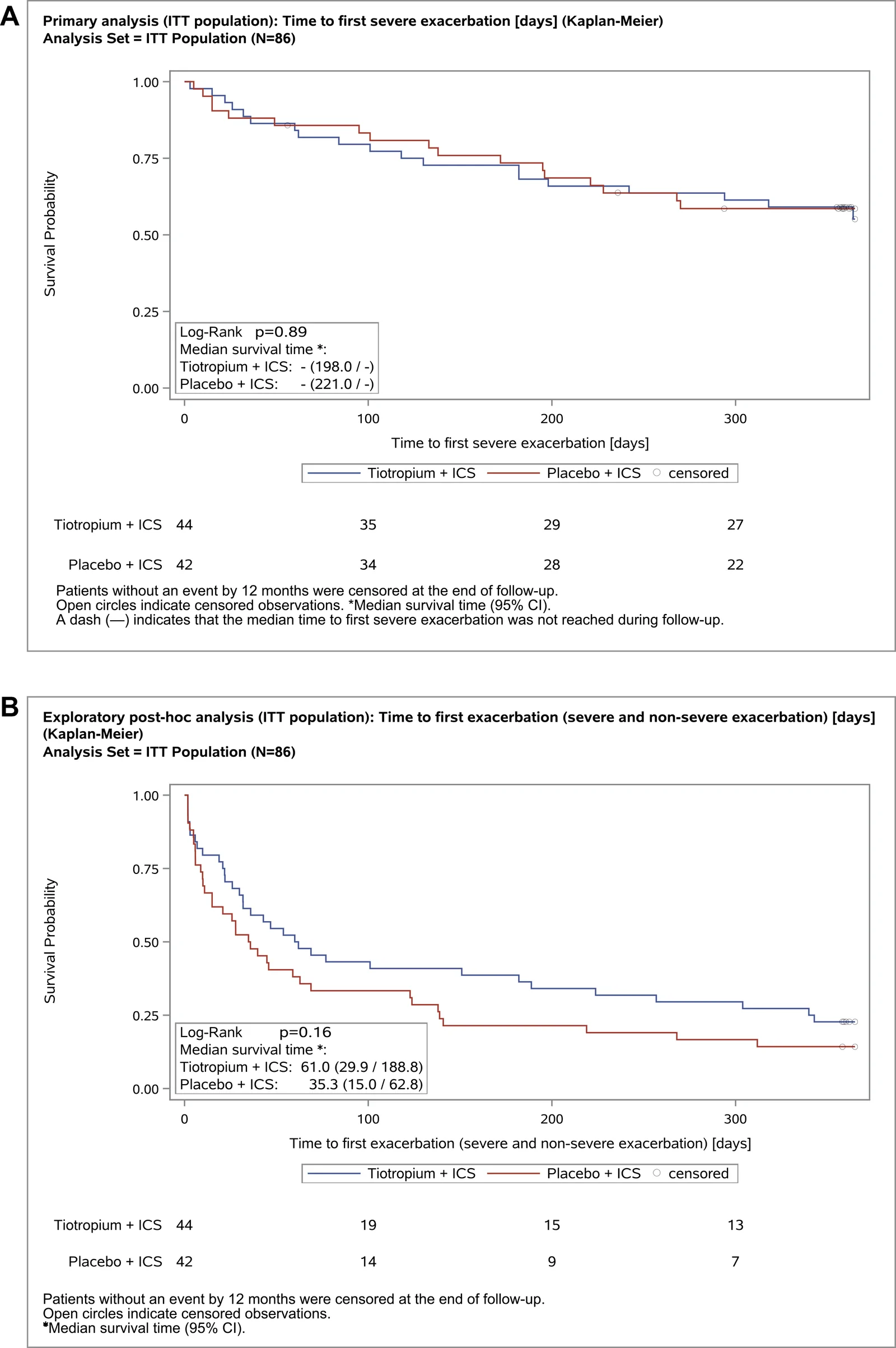
a: Severe exacerbation-free survival time in the ITT population. b: Exacerbation-free survival time in the ITT population.

The primary endpoint, time to first severe exacerbation, did not significantly differ between groups. The HR for tiotropium + ICS vs. placebo + ICS was 0.99 (95% CI: 0.51–1.92; p = 0.98), indicating no treatment effect (Fig. 2a). A Cox regression model for recurrent severe exacerbations in the ITT population (161 observations, 77 events) likewise showed no statistically significant treatment effect (HR: 0.72, 95% CI: 0.46–1.11; p = 0.13).

### Secondary outcomes

Among the prespecified secondary endpoints, no statistically significant differences were observed between treatment groups for the number of severe exacerbations, hospitalisations due to severe exacerbations, systemic corticosteroid rescue, percentage of days with salbutamol rescue medication, percentage of days without asthma symptoms, physician visits, missed daycare days, asthma-related antibiotic use, CGI-C, TRACK, lung function outcomes, biomarker analyses, or subgroup analyses (Table 2 and Supplementary material). Regarding patient-reported outcomes assessed with the PACD, night-time awakenings were less frequent in the tiotropium plus ICS group than in the placebo plus ICS group (median 5% [IQR: 3–10] vs. 9% [9–12]; p = 0.024). The median percentage of days with salbutamol rescue medication was 10.7% (IQR: 3–18) in the tiotropium group and 16.7%^7^, ^8^, ^9^, ^10^, ^11^, ^12^, ^13^, ^14^, ^15^, ^16^, ^17^, ^18^, ^19^, ^20^, ^21^, ^22^, ^23^, ^24^, ^25^, ^26^, ^27^, ^28^, ^29^ in the placebo group (p = 0.10). The median number of physician visits (0.0 [IQR: 0–2] vs. 0.0 [0–4]; p = 0.078) and missed daycare days (17 [IQR: 11–35] vs. 27.5 [15–40]; p = 0.32) did not differ significantly between groups. Spirometry was feasible in only a minority of participants because pulmonary function testing could not be performed in 53 children. Accordingly, lung function analyses were restricted to a small subgroup. Response rates and changes in FEV_1_ did not differ significantly between groups (all p > 0.05).

A negative binomial regression showed no significant difference in rate of mild exacerbations with tiotropium plus ICS compared with placebo plus ICS (Incidence rate ratio (IRR): 0.76, 95% CI: 0.54–1.07; p = 0.12).

Across treatment groups, children aged 1–3 years had a higher rate of mild exacerbations than children aged 4–5 years after adjustment for treatment group and study centre (adjusted IRR: 1.57, 95% CI: 1.11–2.22; p = 0.012). This finding represents an age-related covariate association and not a treatment-by-age interaction.

### Exploratory post-hoc analyses

In a post-hoc analysis combining mild and severe exacerbations, negative binomial regression showed no statistically significant treatment effect (IRR: 0.82, 95% CI: 0.59–1.15; p = 0.25). Younger age remained independently associated with a higher exacerbation rate (IRR: 1.52, 95% CI: 1.08–2.14; p = 0.016). Descriptively, the mean number of total exacerbations per patient-year was 3.4 in the tiotropium group and 4.2 in the placebo group.

A post-hoc Cox regression analysis suggested longer exacerbation-free survival in the tiotropium group (HR: 0.60; p = 0.042). However, the corresponding log-rank test was not statistically significant (p = 0.16) (Fig. 2b).

### Safety analyses

At least one AE was documented in 83 (97%) of all patients (Table 3). A total of 847 AEs were reported (average of 10 per patient). Of these, 397 AEs (47%, average of 9 per patient) occurred in the tiotropium + ICS group and 450 AEs (53%, average of 11 per patient) occurred in the placebo + ICS group. 436 (51%) of all AEs were classified as mild, 329 (39%) as moderate, and 82 (10%) as severe. 39 (48%, average 0.89 per patient) of the severe AEs occurred in the tiotropium + ICS group and 43 (52%, average one per patient) in the placebo + ICS group. None of the severe AEs were assessed as having a positive causal relationship with the study medication. AEs related to asthma or respiratory tract infections occurred frequently, including nasopharyngitis, bronchitis, and pneumonia (Table 3). It is noteworthy that upper respiratory tract infections (1 vs. 8) and pneumonia (9 vs. 17) occurred more frequently in the placebo + ICS group. No new AEs related to tiotropium treatment were observed.

## Discussion

To our knowledge, the TIPP study is the first to assess long-term efficacy of tiotropium over 12 months as add-on treatment to ICS in children with partially controlled or uncontrolled preschool asthma.

The TIPP study showed no benefit of tiotropium over placebo with regard to the primary endpoint, ‘time to first severe exacerbation’. Exploratory caregiver-reported symptom analyses conducted using a novel electronic application showed no significant differences between the treatment groups except for night-time awakenings which were less frequent in the tiotropium plus ICS group than in the placebo plus ICS group. This finding require confirmation in adequately powered trials with appropriate control for multiplicity.

Exploratory biomarker analyses in our cohort showed no evidence of differential treatment response by eosinophil count, total IgE, or allergen sensitisation status; however, these analyses were limited by small subgroup sizes. In older children, tiotropium efficacy has been reported to be largely independent of T2 inflammatory status, suggesting that any response may not be restricted to a T2-high phenotype.^31^

Although preschool children with asthma are particularly prone to severe exacerbations requiring emergency department visits and hospital admissions, the choice of medications for this vulnerable population is very limited.^1^^,^^13^^,^^14^ ICS are considered first-line controller medication; while in older children LABAs are first choice as add-on medication, however, these are not licensed under the age of 4; tiotropium has only been licensed from age 6 years as add-on maintenance to ICS. Preschool asthma is often described as exacerbation-prone, with relatively minor impairments.^32^ However, young children with frequent severe exacerbations are common, and caregivers frequently report relevant coughing and wheezing, as well as absences from day care, particularly during winter.^33^^,^^34^ The high symptom burden in the selected TIPP cohort was recently confirmed in our first report of the enrolled patients.^4^

Tiotropium is approved as an add-on maintenance bronchodilator treatment for patients aged 6–17 years with asthma who remain symptomatic despite standard therapy with ICS. In this age group, tiotropium has been shown to significantly improve FEV_1_ compared with placebo, and to reduce rescue bronchodilator use, nocturnal awakenings, and the frequency and severity of exacerbations.^21^^,^^22^^,^^35^^,^^36^ Data on tiotropium in preschool asthma are limited. Only one prospective study has evaluated tiotropium as adjunctive therapy in preschool children with persistent asthma symptoms.^23^ This 12-week trial showed no differences in the safety profile between groups receiving 5 μg tiotropium, 2.5 μg tiotropium, or placebo.^23^

In addition, a retrospective report from our group showed that tiotropium was effective in severe preschool asthma, with significantly fewer asthma episodes, antibiotic prescriptions, and physician visits after tiotropium was added to ICS.^24^ Recently, Kotaniemi-Syrjänen et al.^25^ demonstrated the bronchodilator potency of tiotropium in an open-label controlled study of episodic wheezers aged 6–35 months: the proportion of episode-free days was higher in children receiving intermittent tiotropium (median 97% [IQR: 93–99%]) than in those receiving intermittent fluticasone propionate (IQR: 87% [78–93%]; p = 0.002) or as-needed albuterol sulphate alone (IQR: 88% [79–95%]; p = 0.003).

These findings are relevant for affected children, their parents, and treating physicians. In addition, tiotropium inhalation is considered a safe long-term treatment in asthma. A total of 847 AEs were reported in our study. The most frequently reported AEs were asthma exacerbations, acute bronchitis, and nasopharyngitis. The distribution and pattern of AEs were largely similar across the treatment groups, and no new safety signals associated with tiotropium were identified. Overall, our safety findings are consistent with those of previous paediatric studies.^21^^,^^22^ Spirometry was performed in patients aged ≥ 4 years who were able to perform technically acceptable and reproducible PFTs. Lung function analyses were therefore limited to a small subgroup and should be interpreted cautiously. Interestingly, at visit 4 (week 24), the tiotropium group showed an average increase in FEV_1_ of 131 mL from baseline, a clinically relevant improvement that is consistent with findings in older children.^21^^,^^36^ Although these numerical differences favoured tiotropium, none reached statistical significance.

This study has several important limitations. First, recruitment fell substantially short of the original target, leaving the trial underpowered for the primary endpoint and limiting the precision of secondary and subgroup analyses. Second, no multiplicity adjustment was applied; therefore, nominally significant secondary findings should be interpreted as exploratory. Third, preschool asthma is a heterogeneous clinical entity, and despite protocol-defined eligibility criteria, phenotypic heterogeneity may have diluted treatment effects. Additionally, stratification factors such as gender and the severity of the history of exacerbations might have led to a better balance in a larger study; however, this was not feasible in this study given the final sample size and the multicentre recruitment. Fourth, background controller therapy was heterogeneous, although treatment groups were descriptively similar at baseline. Fifth, lung function data were available only in a limited subgroup of older children able to perform acceptable manoeuvres. Finally, the sample size was insufficient to draw firm conclusions regarding rare AEs.

The authors see various reasons for the great difficulties in recruiting patients. One main reason is certainly the largely inconsistent nomenclature and diagnosis of asthma in preschool children. The ongoing debate about whether asthma should be diagnosed in preschool children at all plays a decisive role in this. Due to the inconsistent nomenclature (virus-induced wheezing/wheezing caused by multiple triggers/asthma), even in large centres, some patients are not adequately diagnosed and treated. A significant proportion of young children with asthma are diagnosed with recurrent obstructive bronchitis or recurrent wheezing and are only treated by their family doctor during acute exacerbations, rather than in specialised centres. In addition, the standard of medical care in Germany is high and is fully covered by public health insurance resulting in scepticism towards clinical studies especially in very young children. A further extension of the study was not possible for cost reasons and due to regulatory requirements of the Federal Ministry of Education and Research.

In conclusion, in this randomised trial in preschool children with high-risk asthma despite ICS treatment, tiotropium did not reduce the risk of a severe asthma exacerbation but was well tolerated.

## Contributors

SZ: Writing—original draft, Writing—review & editing, Conceptualisation, Data curation, Formal Analysis, Funding acquisition, Investigation, Methodology, Project administration, Resources, Supervision, Validation, Visualisation, Accessed and verified the data. TN: Data curation, Investigation, Methodology, Supervision, Validation, Writing—review & editing. CG: Investigation, Writing—review & editing. DS: Investigation, Writing—review & editing. FS: Investigation, Writing—review & editing. FP: Investigation, Writing—review & editing. FZ: Investigation, Writing—review & editing. BS: Investigation, Writing—review & editing. CL: Investigation, Writing—review & editing. MD: Investigation, Writing—review & editing. JT: Investigation, Software, Supervision, Writing—review & editing. HD: Investigation, Supervision, Writing—review & editing. SL: Investigation, Writing—review & editing. EH: Investigation, Writing—review & editing. CV: Investigation, Writing—review & editing. MG: Investigation, Writing—review & editing. MW: Investigation, Writing—review & editing. RS: Conceptualisation, Investigation, Methodology, Resources, Writing—review & editing. LS: Conceptualisation, Data curation, Funding acquisition, Methodology, Project administration, Software, Supervision, Validation, Visualisation, Writing—original draft. MG: Formal Analysis, Investigation, Writing—review & editing, JW: Conceptualisation, Data curation, Formal Analysis, Methodology, Software, Validation, Visualisation, Accessed and verified the data, Writing—original draft. NW: Data curation, Project administration, Validation, Supervision, Methodology, Writing—review & editing. The corresponding author confirms that he had full access to all the data in the study and had final responsibility for the decision to submit for publication.

## Data sharing statement

The raw data supporting the conclusions of this article will be made available by the authors, without undue reservation.

## Declaration of interests

SZ reports grants and personal fees from Allergy Therapeutics, Engelhard-Arzneimittel GmbH, Novartis GmbH, Boehringer Ingelheim, IMS HEALTH GmbH & Co. OHG, Stallergen, AstraZeneca, Sanofi/Pasteur, and Erydel outside the submitted work.

MG reports personal lecture honoraria from ALK-Abelló, AstraZeneca, Berlin Chemie, Nestlé, OM-Pharma, and Sanofi-Regeneron; participation on a DSMB or advisory board, paid to institution (ALK-Abelló); leadership or fiduciary role (President: European Respiratory Society, ERS 2023/2024). FP reports payments or honoraria from Sanofi-Regeneron, Vertex Pharmaceuticals, ALK-Abelló, Takeda Pharma, and Biocryst; support for attending meetings and/or travel (BioCryst); participation on a DSMB or advisory board (Takeda Pharma, Sanofi-Regeneron). FS reports honoraria for lectures and invitations to scientific congresses from the German Society for Paediatric Allergology and Environmental Medicine (GPAU), and the Arbeitsgemeinschaft Pädiatrische Allergologie und Pneumologie Süd e.V (AGPAS); support for travel (AGPAS). CV reports a research grant to institution from Boehringer Ingelheim for an IIT; payment or honoraria for lectures from Orion Pharma and AstraZeneca. MG reports consulting fees from GSK; payment or honoraria from AstraZeneca; participation on a DSMB or advisory board (GSK); leadership or fiduciary role in societies (Society for Paediatric Allergology, GPA). RG reports all support for the present manuscript to institution from the German Centre for Lung Research and the Clinician Scientist Programme TITUS (Else Kröner-Fresenius Kolleg). CL reports all support for the present manuscript to institution from the German Centre for Lung Research and the Clinician Scientist Programme TITUS (Else Kröner-Fresenius Kolleg). BS reports all support for the present manuscript from the German Centre for Lung Research and the German Research Foundation; grants or contracts form the German Federal Ministry of Education and Research; consulting fees from GSK, Novartis, AstraZeneca and Sanofi; payments or honoraria from Sanofi; participation on a DSMB or advisory board (Sanofi). RS reports grants or contracts to institution from the Starke Lunge Foundation—miR-Solution. EH reports all support for the present manuscript to institution from the Network University Medicine (NUM), and the National Pandemic Cohort (NAPKON); grants or contracts from the German Federal Ministry of Education and Research to institution; grants and contracts from the German Ministry of Health; consulting fees from ALK-Abelló, AstraZeneca and Sanofi; payments or honoraria from ALK-Abelló, AstraZeneca, Sanofi, Stallergenes Greer, Novartis, AImmune, and Milupa-Danone; leadership or fiduciary roles in societies (President: German Society for Allergy and Clinical Immunology, DGAKI; head of asthma section: Society for Paediatric Pneumology, GPP; president: German Asthma Net, GAN; board member: Society for Paediatric Allergology, GPA and German Society for Paediatrics); participation on a DSMB or advisory board (AstraZeneca, Sanofi). All other authors declare no competing interests.

## Appendix A Supplementary data

Supplementary material

TIPP_Protocol_V4.0

TIPP_SAP_V1.0
