# Supplementary material for: Efficacy and safety of tiotropium in partial and uncontrolled preschool asthma (TIPP): a phase III, multicentre, randomised, double-blind, placebo-controlled trial
Source: eClinicalMedicine. 2026 Sep 4;99:104172. doi: 10.1016/j.eclinm.2026.104172 (PMC13571877; doi:10.1016/j.eclinm.2026.104172)
Supplement: Supplementary file 2 [file mmc3.pdf]

A prospective multicenter placebo-controlled trial to study the efficacy and safety of Tiotropium in preventing severe asthma exacerbations in partial and uncontrolled preschool asthma.

TIPP-Study

Statistical Analysis Plan

Version 1.0 07.08.2025

Prof. Dr. med Stefan Zielen	______________________
Coordinating investigator	Date, Signature
Julia Wosniok	______________________
Statistician	Date, Signature

1 Study background

Asthma is the most common chronic disease in children, imposing a high lifetime burden on individuals, their caregivers, and healthcare systems (Ferrante et al., 2018; European Respiratory Society, 2019). The prevalence of childhood asthma has increased over the last 20 years, most likely due to a greater awareness of this condition and changes in diagnostic practice. In Germany, parent-reported asthma prevalence in preschool children (< 6 years) ranged from 2.6 to 2.8% (Weber et al., 2016).

As in adults, a considerable proportion of asthma in children is not well controlled by inhaled corticosteroids (ICS), which represents a significant healthcare concern (Liu et al., 2010). Failure to control asthma has a negative impact on patients' quality of life, and increases the risk of future exacerbations, with associated increased requirement for healthcare utilization and costs (Guilbert et al., 2011; Haselkorn et al., 2009).

Optimizing asthma management among preschool children is an unmet need since this age group experiences disproportional morbidity and health care utilization compared to school-age children with asthma (Akinbami et al., 2009; Akinbami et al., 2006; Beigelman et al., 2017). At present, there are insufficient data to recommend additional controller therapies, such as combinations of ICS with LABAs or LAMAs in this age group (Global Initiative for Asthma, 2019).

1.1 Goal of the study

The primary objective is to evaluate whether the addition of Tiotropium via Respimat® to ICS prolongs the time to first severe asthma exacerbation (defined by hospitalization and/or three consecutive days of oral or parenteral systemic steroid intake or one day of rectal prednisolone application (Rectodelt®).

The secondary objective is to evaluate whether addition of Tiotropium via Respimat® to ICS reduces health utilization (hospitalizations, physician visits, antibiotic use) in partial or uncontrolled preschool asthma.

1.2 Study design

TIPP is a prospective randomized, double-blind, placebo-controlled, parallel-group trial (a non-commercial trial, clinical phase III) to evaluate the safety and efficacy of Tiotropium inhalation solution (2.5 µg) administered once daily via Respimat® Inhaler for 52 weeks to prevent severe asthma exacerbations in patients 1–5 years with uncontrolled or partially controlled preschool asthma and at least one severe asthma exacerbation requiring hospitalization and/or was treated with 2 courses of systemic steroids in the last 24 months before visit 1 (three days of oral systemic steroids or one day of rectal prednisolone).

2 Analysis Populations

2.1 Definitions

All patients whose parents/guardians signed informed consent are considered as enrolled.

All randomized patients will be included in the intention-to-treat (ITT) population. This population is the primary analysis population. Within the ITT population analyses patients will be assigned to the treatment to which they were randomized.

To be eligible for the per-protocol (PP) population, patients must fulfill at least the following criteria:

- No major protocol violations (see section 2.3)
- No concomitant treatment with oral antibiotics longer than 14 consecutive days or any monoclonal antibody at any time

• Treatment compliance of at least 80% referring to the visits that were attended.

The safety population comprises all patients who received at least one dose of trial treatment. In analyses of the safety population patients will be assigned to the treatment which they actually received. The analysis populations will be defined before unblinding of the study.

Special cases:

- Patient 02/01 received the wrong dose by mistake (parents' fault) and prematurely discontinued study medication. This patient is to be excluded from the PP population.

- Patient 11/01 received a dose that was too low and therefore has to be excluded from the PP population.

- Patient 01/15 accidentally received and used one inhaler with random number 015 instead of number 010 (after the patient who actually received inhaler 015 had already completed the study). After unblinding, it has to be checked, if inhaler 015 belongs to the same study arm as inhaler 010. If yes, the patient is still eligible for the per protocol analysis, if not, s/he has to be excluded from the PP population. Independent of this the patient will be analyzed in the ITT population for the randomized treatment.

- Patient 01/43 accidentally received Tiotropium in addition to the study treatment and therefor will be excluded from the PP population.

2.2 Areas of application

The primary population for the analyses of efficacy is the ITT population.

The primary will be repeated for the PP population.

Safety analyses will be performed in the safety population.

2.3 Major Protocol Violations

- Violation of inclusion or exclusion criteria
- Patient did not receive the treatment s/he was assigned to by randomization
- Intake of not permitted medication as defined in the protocol

3 Study centres

Data from 12 study centres will be available for the analysis. No center effects will be explored as the number of patients per center is too small.

4 Analysis variables

4.1 Patient disposition

4.1.1 Patient disposition

The following variables of patient disposition will be analysed:

- Informed consent (Y/N)
- Populations (ITT, PP, Safety)
- In- and exclusion criteria
- Derived: Number of patients taking part for each study visit (if visit date of patient is available)

The following data are needed for the creation of a CONSORT flow chart:

- Number of patients screened
- Number of patients discontinued between screening and enrolment including reason
- Number of patients enrolled (signed informed consent)
- Number of patients discontinued between enrolment and randomzation including reason
- Number of patients randomized
- Number of patients treated (by study arm)
- Number of patients discontinued treatment including reason (by study arm)
- Number of patients finished treatment regularly (by study arm)

4.2 Demography and Baseline Characteristics

4.2.1 Demography

The following variables of demography will be analysed:

- age
- sex (Female/ Male/ Unknown)
- race (White, Asian, Black, Other)

4.2.2 Baseline Characteristics

4.2.2.1 Current diagnosis

The following variables of current diagnosis will be analysed:

- Derived: Time since asthma diagnosis (screening date- date of onset of asthma+1)
- Household pet (dog, cat, guinea pig, mouse, rabbit, hamster, other [specify], no household pet; Y/N)
- Passive smoking (Y/N)
- Passive smoker: Number of Caregivers (1 smoker, 2 smokers, more than 2 smokers)

4.2.2.2 Health resource utilization (between V1 and V2)

The following variables of health resource utilization will be analysed:

- Hospitalisation intensive care unit (Y/ N)
- Hospitalisation intensive care unit (Days)
- Hospitalisation normal station (Y/ N)
- Hospitalisation normal station (Days)
- Appointment Emergency Doctor/ Emergency room? (Y/N)
- Appointment Emergency Doctor/ Emergency room? (Days)
- Paediatrician/ Family Doctor Contact? (Y/ N)
- Paediatrician/ Family Doctor Contact? (Days)
- Other Contact With The Health Service? (Y/ N)
- Other Contact With The Health Service? (Days)
- Number Of Missed Days in Daycare (Days)

These parameters are evaluated at different points in time. Baseline values of these parameters will be analyzed and presented together with data evaluated at further visits during the course of the study.

4.2.2.3 Vital signs, physical examination, GINA

The following variables of vital signs/ physical examination will be analysed:

- Vital signs (Height [cm], Weight [kg], Heart rate [Bpm], Body temperature [°C}, Systolic blood pressure [mmHg], Diastolic blood pressure [mmHg])
- Result of physical examination (No findings/ Abnormal findings, NOT clinically significant/ Abnormal findings, clinically significant, not done)

- GINA (Daytime symptoms (Y/ N), Limitation of activities (Y/ N), Nocturnal symptoms/ awakening (Y/ N), Need for reliever/ rescue treatment (Y/ N), Level of asthma control (well controlled, Partly controlled, uncontrolled))

  These parameters are evaluated at different points in time. Baseline values of these parameters will be analyzed and presented together with data evaluated at further visits during the course of the study.

4.2.2.4 Laboratory testing

The following variables of laboratory testing will be analysed:

- Laboratory Not Feasible
- Blood count (White Blood Cells [Tsd/ µL], Red Blood Cells [Mio/ µL], Hemoglobin [g/dL], Hematocrit [%], MCV [fl], MCH [pg], MCHC [g/dL], Platelets [10^9/L], Absolute neutrophil count [10^3/µL], Lymphocytes absolute [10^3/µL], Monocytes [10^3/µL], Eosinophils [10^3/µL], Basophils [10^3/µL]-)
- Liver Enzymes (ALT [U/L], AST [U/L], Alkaline phosphatase [U/L],)
- Kidney function (Total urea [mg/dL], Creatinine [mg/dL])
- Laboratory clinically significant (Y/ N)

These parameters are evaluated at different points in time. Baseline values of these parameters will be analyzed and presented together with data evaluated at further visits during the course of the study.

4.2.2.5 Allergy Diagnostic/ specific IgE

The following variables of allergy diagnostic/ specific IgE will be analysed:

- Total IgE [kU/L]
- Total IgE >= 25 kU/L (Y/N)
- Total IgE >= 100 kU/L (Y/N)
- EOS >=300 µL
- Specific IgE [kU/L] as well as Specific IgE >=0.75 kU/L (Y/N) for the following allergens:
  - Pollen allergens all >=0.75 kU/L (Y/N)
    - Birch
    - Cladosporium
    - Grass
  - Perennial allergens all >=0.75 kU/L (Y/N)
    - Alternaria
    - Dermatophagoides pteronyssinus
    - Cat
    - Dog
  - Food allergens all >=0.75 kU/L (Y/N)
    - Hen’s egg
    - Cow’s milk
    - Peanut

These parameters are evaluated at different points in time. Baseline values of these parameters will be analyzed and presented together with data evaluated at further visits during the course of the study.

4.2.2.6 Spirometry/ Impulse oscillometry

The following variables of spirometry/ Impulse oscillometry will be analysed:

- Oscillometry:
  - Oscillometry not feasible
  - R5 [%]
  - R5 (Z-Score)

- X5 [Kpa*s*l-1]
- X5 (Z-Score)
- Dr5-20 (Difference Between Actual And Target Value) [Kpa*s*l-1]
- AX ([Kpa/L]

- Spirometry:
  - Spirometry not feasible
  - FVC [L]
  - FVC [%pred}]
  - FVC [z-score]
  - FEV1 [L]
  - FEV1 [%pred]
  - FEV1 [z-score]
  - FEV1%FVC

These parameters are evaluated at different points in time. Baseline values of these parameters will be analyzed and presented together with data evaluated at further visits during the course of the study

4.2.2.7 Track test

The following variables of track test will be analysed:

- Track not feasible
- Track Question 1 (4 or more times a week, 2 or 3 times a week, Once every week, Once or twice, ot at all)
- Track Question 2 (4 or more times a week, 2 or 3 times a week, Once every week, Once or twice, Not at all)
- Track Question 3 (Extremely, quite a lot, moderately, slightly, not at all)
- Track Question 4 (4 or more times a week, 2 or 3 times a week, Once every week, Once or twice, Not at all)
- Track Question 5 (4 or more times, 3 times, twice, once, never)
- Track total

These parameters are evaluated at different points in time. Baseline values of these parameters will be analyzed and presented together with data evaluated at further visits during the course of the study

*4.2.2.8 Patient Diary (PACD-*Pediatric asthma caregiver diary*)*

For evaluations of baseline PACD, data of the first 28 days after V1 will be used, independent from the month of recruitment. Data from after V2 (see secondary endpoints) will be evaluated only for the months September to (end of) April, as the PACD was stopped for 4 months in summer.
In case of missing data in the months September to April, it is assumed that the children experienced no symptoms and the data of the diary will be imputed accordingly. Missing data in the 28 days after V1 will also be imputed independent from the month.

The following variables of the patient diary will be analysed.

- Derived: Number and percentage of days with asthma symptoms between V1 and V2 (Day 1-28) assessed by TIPP diary app, derived from questions 1-3, 5,7, 10-13 [n, %]
- Derived: Number and percentage of days without asthma symptoms between V1 and V2 (Day 1-28) assessed by TIPP diary app, derived from questions 1-3, 5,7, 10-13 [n, %]
- Derived: Number and percentage of night-time awakenings due to asthma symptoms between V1 and V2 (Day 1-28) assessed by TIPP diary app, derived from question 2 [n, %]

- Derived: Number and percentage of missed days in day care between V1 and V2 (Day 1-28) assessed by TIPP diary app, derived from question 10 [n, %]
- Derived: Number and percentage of asthma-related events defined by use of systemic corticosteroids between V1 and V2 (Day 1-28) assessed by TIPP diary app, derived from question 12 [n, %]

Derived: Number and percentage of asthma-related events defined by use of antibiotics between V1 and V2 (Day 1-28) assessed by TIPP diary app, derived from question 13 [n, %]

4.3 Extent of exposure and compliance

The following variables of the patient diary will be analysed:

- Derived: Extent of exposure [days] (end of study-date of randomization+1)
- Compliance by study visit (Y/N)
- Correct intake according to protocol by visit (Y/N)

4.4 Primary Variable

The primary variable is the time to first severe exacerbation (from start of treatment) defined by hospitalization and/or at least 3 days of systemic steroids or one day of rectal prednisolone (Rectodelt®). Severe exacerbations were chosen as primary outcome because they are patient-important outcomes that have significant consequences for children, caregivers, and the health care system.

4.5 Secondary Variables

4.5.1 Effectiveness

- Number of severe exacerbations
- Exacerbation-free survival time defined as the time from randomization until the first severe exacerbation
- Percentage of patients requiring systemic corticosteroid rescue for asthma during the 52-week treatment period (information to be taken from concomitant medication page)
- Number of hospitalizations (severe exacerbation)
- Percentage of days and episodes of >3 days with use of PRN salbutamol rescue medication during 52-week treatment period (non-severe exacerbation) (information to be taken from concomitant medication page)
- Percentage of days without asthma symptoms assessed by TIPP diary App (PACD).
  - A day without asthma symptoms is defined as a day during which the patient experienced no asthma symptoms (daytime and overnight; question 1, 2, 5, 6 , did not use rescue medication (salbutamol) (question 3 and 7), and had no asthma exacerbation/worsening requiring systemic corticosteroids, an unscheduled visit to a doctor's office, or hospital (question 11).
- Percentage of night-time awakenings after start of treatment due to asthma symptoms as assessed by the TIPP diary App
- Health utilization defined as number of physician visits during treatment
- Number of missed days in daycare (information to be taken from eCRF)
- Number of asthma-related events defined by use of antibiotics (information to be taken from concomitant medication page)
- Evaluation of TRACK at V1, at V4 and at end of treatment
- CGI-C at end of treatment

- **Additional secondary endpoints for children aged 4 years and older (**only applicable for patients able to perform technically acceptable (and reproducible) pulmonary function tests):

- The forced expiratory volume in one second (FEV1) and the forced vital capacity (FVC) measured 30 minutes post IMP will be compared to pre-dosing FEV1 and FVC values at visit 2.
- Trough FEV1 and trough FVC response will be determined at visit 3, visit 4, visit 5 and visit 7. Trough FEV1/ FVC is defined as the FEV1/ FVC measured approximately 24 hours after the last IMP inhalation. Response is defined as a 10% improvement in FEV1/ FVC, respectively, compared to baseline (visit 2, pre-dosing).
  Also the difference to baseline (visit 2, pre-dosing) for FEV1/ FVC for visit 3, visit 4, visit 5 and visit 7 will be presented.
- Potential biomarkers for treatment response (Eosinophils, IgE, specific IgE to common allergens: birch, grass, *Dermatophagoides pteronyssinus*, *Alternaria*, *Cladosporium*, cat, dog, hen's egg, cow's milk and peanut)

4.5.2 Exploratory endpoints

For exploratory analyses miRNA profiles obtained by next generation sequencing will be analyzed. These analyses will be carried out at a later timepoint in order to publish them in a scientific journal (not mandatory for the study report). miRNA profiles will be compared for the following groups (changes possible):

- Children with preschool asthma compared to healthy children (external control group)
- Allergic vs. non-allergic patients
- Patients treated with ICS+ Tiotropium vs. patients treated with ICS+ placebo
- Patients with none/ few exacerbations vs. patients with a high number of exacerbations

4.5.3 Safety Variables

4.5.3.1 Adverse events

AEs will be coded according to MedDRA terminology. Detailed information collected for each AE will include a description of the event, duration, whether the AE was serious, intensity, relationship to trial drug, action taken and clinical outcome. Summary tables will present the number of patients observed with AEs by MedDRA System Organ Class and Preferred Term and corresponding percentages. Additional subcategories will be based on event intensity and relationship to trial drug.
A patient listing of all AEs will be prepared.

4.5.3.2 Laboratory data

Laboratory data as described in section 4.2.2.4 were collected at different timepoints in the course of the study, including the assessment if the parameter is clinically significant.

4.5.3.3 Prior and Concomitant diseases/medical history

All concomitant diseases/medical history reported in the eCRF are collected with term, start and stop date and are coded with MedDRA terminology.

4.5.3.4 Prior and Concomitant medication

All concomitant medication reported in the eCRF are collected with term, indication, dose, route, start and stop date and are coded by ATC classification.

Patients will be grouped into the following exclusive categories concerning ICS maintenance treatment before randomization. Each patient can be assigned to only one category:

- ICS mono *(Fluticason (Flutide®))*
- ICS + LABA *(Fluticason + Sameterol (Viani mite®))*
- *ICS (Fluticason (Flutide®))* + LTRA *(Montelukast)*
- ICS + LABA *(Fluticason + Sameterol (Viani mite®))*+ LTRA *(Montelukast)*

4.5.3.5 Vital parameters

Vital parameters as described in section 4.2.2.3 were collected at different timepoints in the course of the study.

5 Handling missing values and outliers

5.1 Missing values

Diary (PACD)
In case of missing data, it is assumed that children experienced no symptoms; the data will be imputed accordingly (no asthma symptoms, no salbutamol use).

For the first 28 days after V1 and before V2: Missing data will be imputed independent from the month.
Visits after randomisation: Missing data will be imputed for the months September to April.

5.2 Outliers

No methods for outlier detection will be applied. In case of obvious implausible data, the data will be set to missing.

6 Statistical analysis

The default summary statistics for quantitative variables will be the number of observations (n), mean, standard deviation (SD), median, first quartile (Q1), third quartile (Q3), minimum and maximum for those patients with data available.
For qualitative variables, the number (n) and percentage (%) of patients with non-missing data per category will be the default summary presentation. If appropriate, the number of missing values will be displayed as a “Missing” category.
All variables will be displayed by treatment group and with a total column.
Listings of all AEs and laboratory measures will be provided.
Tables, listings and figures will be provided in English language.

6.1 Patient disposition

Variables of patient disposition will be presented by absolute and relative frequencies and if possible, by study visit and study arm.

6.2 Demography and Baseline characteristics

Continuous and quasi-continuous data will be displayed by the sample characteristics N (number of non-missing observations), mean, standard deviation, minimum, median, maximum and number of missing observations. Categorical data will be presented by absolute and relative frequencies.

6.3 Extent of exposure and Compliance

Continuous and quasi-continuous data will be displayed by the sample characteristics N (number of non-missing observations), mean, standard deviation, minimum, median, maximum and number of missing observations. Categorical data will be presented by absolute and relative frequencies.

6.4 Concomitant diseases/medical history

All concomitant diseases/medical history reported in the eCRF are collected with term, start and stop date and are coded with MedDRA terminology.

6.5 Prior and Concomitant medication

All concomitant medication reported in the eCRF are collected with term, indication, dose, route, start and stop date and are coded by ATC classification.

6.6 Primary analysis

The primary analysis is the comparison of time to first severe exacerbation within 12 months of therapy between groups. This means especially that observations after 12 months will be censored. Dropouts because of lack of efficacy will be considered as an exacerbation (for programming: in case of irregular end of study → other → lack of efficacy). All other dropouts will be censored. If an exacerbation starts mildly and becomes severe, the first day of the severe exacerbation is to be counted. Also, exacerbations with a break of at least one day will be counted as 2 separate exacerbations.
The comparison will be done on a two-sided significance level of α = 5%.
The primary hypotheses are:

H_0: $h_E/h_C = 1$ versus H_1: $h_E/h_C \neq 1$

Where h_E, h_C are the hazards in the experimental treatment group and the control treatment group respectively.

The time to first severe exacerbation will be analyzed by Cox regression with factor treatment group and the covariates age group (1-3 yrs vs. 4-5 yrs) and center (center 1 [Frankfurt] vs. other centers) assuming a constant hazard ratio over time. The constant hazard ratio assumption will be checked by Schoenfeld residuals. The primary analyses will be performed in the ITT population. Treatment differences will be displayed by estimates of the hazard ratios and 95% confidence intervals.

The following sensitivity analyses will be performed. The primary analysis Tiotropium + at least ICS versus placebo + ICS will be repeated additionally adjusted for LABA and LTRA use. This analysis is not primary since LABA use is not treatment independent.

A time to recurrent event analysis is also performed. Since a substantial correlation between the events is expected a Prentice Williams and Peterson (PWP) model with the age group and center will be employed.

Additionally, a Kaplan-Meier analysis including a Kaplan-Meier plot will be done.

All analyses will be repeated for the per protocol population.

6.7 Secondary analyses

6.7.1 Effectiveness

The number of severe exacerbations, the number of hospitalizations as well as the number of asthma-related events defined by use of antibiotics will be assessed by negative binomial regression with time within study (from start of treatment) as offset variable.

Exacerbation-free survival time will be analyzed by Cox regression.

Further secondary endpoints will be assessed by chi-square or Fisher's exact test for categorical variables or t-test or Mann-Whitney-U-test for continuous variables.

All tests are two-sided, and a p-value of less than 0.05 is considered to indicate statistical significance. Treatment differences will be displayed by hazard ratios, odds ratios, and 95% confidence intervals.

6.7.2 Exploratory endpoints

For the analysis of the miRNA profiles, appropriate statistical methods will be applied, contingent upon the final format and characteristics of the data.

6.8 Safety

6.8.1 Adverse Events

All adverse events as reported in the eCRF are displayed individually per patient in listings inclusive MedDRA coded terms.
Only treatment-emergent adverse events occurring in subjects administered study treatment will be evaluated.
AEs will be coded according to MedDRA terminology. Detailed information collected for each AE will include: A description of the event, duration, whether the AE was serious, intensity, relationship to trial drug and clinical outcome.
Summary tables will present the number of subjects observed with AEs by MedDRA System Organ Class and Preferred Term and corresponding percentages.

A summary table will be produced showing the number of patients with
- any AEs
- any SAEs
- any fatal AEs
- any severe AEs
- any related AEs
- any related severe AEs
- any related serious AEs
- any fatal related AEs
- Any exacerbation
- Any severe Exacerbation
- Any asthma-related event

A subject listing of all AEs will be prepared.

6.8.1.1 Laboratory parameters

Structured listings will be prepared for each laboratory measure to permit review of the data per patient as they progress on treatment.
Summary tables will be prepared to examine the changes of laboratory measures (including changes to baseline) over time.

6.8.1.2 Vital Signs

Continuous and quasi-continuous data will be displayed by the sample characteristics N (number of non-missing observations), mean, standard deviation, minimum, median, maximum and number of missing observations. Categorical data will be presented by absolute and relative frequencies.

6.9 Subgroup analyses

The following subgroups will be defined:
- male vs. female patients
- Allergic vs. non-allergic patients.
  - Allergic patients is defined as measured sIgE≥0.75 K/UL to any of the tested allergens like birch, grass, mites, Alternaria, Cladosporium, cat, dog, milk, egg and peanut.
  - non-allergic patients are defined as the absence of sIgE levels >0.75 KU/L.

For the subgroup analyses, the primary analysis (Cox-Regression) will be repeated including the subgroup. Also, Kaplan-Meier plots will be created.

6.10 Interim analyses

No interim analyses are planned.

7 Software

SAS Version 9.4 will be used for all analyses.
