# Supplementary material for: Efficacy and safety of tiotropium in partial and uncontrolled preschool asthma (TIPP): a phase III, multicentre, randomised, double-blind, placebo-controlled trial
Source: eClinicalMedicine. 2026 Sep 4;99:104172. doi: 10.1016/j.eclinm.2026.104172 (PMC13571877; doi:10.1016/j.eclinm.2026.104172)
Supplement: Supplementary file 3 [file mmc1.docx]

**Supplementary Material**

Efficacy and safety of Tiotropium in partial and uncontrolled preschool asthma (TIPP): a phase III, multicentre, randomised, double-blind, placebo-controlled trial

Prof. Stefan Zielen MD ^1,2^, Nadine Wollscheid PhD ^3^, Tanja Nickolay MD ^3^, Caroline Grimmel MD ^4^, David Scheele MD ^1^, Franziska Sattler MD ^5^, Freerk Prenzel MD ^6^, Michael Lorenz MD ^7^, Prof. Bianca Schaub MD ^5^, Prof. Christiane Lex MD ^8^, Franziska Stieglitz MD 9, Jordis Trischler MD ^1^, Helena Donath MD ^1^, Prof. Susanne Lau MD ^10^, Prof. Eckard Hamelmann MD ^11^, Prof. Christian Vogelberg MD ^12^, Markus Gerstlauer MD ^13^, Ruth Grychtol MD ^14^, Prof. Ralf Schubert PhD ^1^, Prof. Monika Gappa MD ^4^, Lukas Schollenberger PhD ^3^, Julia Wosniok ^3^.

Department for Children and Adolescents, Goethe-University Frankfurt, Frankfurt ^1^, and

Institute of Respiratory Research, Medaimun GmbH, 60596 Frankfurt, Germany ^2^,

Interdisciplinary Centre for Clinical Trials (IZKS), University Medical Centre Mainz, Mainz, Germany ^3^, Children's Hospital, Evangelisches Krankenhaus Düsseldorf, Düsseldorf, Germany ^4^,

Paediatric Allergology, Department of Paediatrics, Dr. von Hauner Children's University Hospital, LMU Munich-Member of the German Centre for Lung Research-DZL-LMU Munich, Munich, Germany ^5^, University of Leipzig, Medical Centre, Department of Paediatrics, Leipzig, Germany ^6^,

Department of Paediatrics, Jena University Hospital, Friedrich-Schiller-University, Jena, Germany ^7^ , Department of Paediatrics and Adolescent Medicine, Division of Paediatric Cardiology and Intensive Care, University Medicine Göttingen, Göttingen, Germany ^8^,

Kinderlungen-Facharzt, Gemeinschaftspraxis in Mannheim, Mannheim, Germany ^9^_,_

Department of Respiratory Medicine, Immunology and Critical Care Medicine, Charité Universitätsmedizin Berlin, Berlin, Germany ^10^,

Department of Paediatrics, Children's Centre Bethel, University Medicine, OWL, University Bielefeld, Bielefeld, Germany ^11^,

Department of Paediatrics, Faculty of Medicine and University Hospital Carl Gustav Carus, Technische Universität Dresden, Dresden, Germany ^12^,

Kinderklinik, Universitätsklinikum Augsburg, Germany ^13^,

Department of Paediatric Pneumology, Allergology, Neonatology, Hannover Medical School, Hannover, Biomedical Research in Endstage and Obstructive Lung Disease (BREATH), Member of the German Centre for Lung Research (DZL), Hannover, Germany ^14^.

Corresponding author:

Prof. Dr. Stefan Zielen, a.D.

Department for Children and Adolescents

Division of Pneumology, Allergology, Infectious Diseases and Gastroenterology

Goethe-University Frankfurt ^1^

Theodor-Stern-Kai 7

60590 Frankfurt

and Institute of Respiratory Research ^2^

Medaimun GmbH

60596 Frankfurt

Germany

**Contents**

[Supplement Table 1: Overview of TIPP study populations.	3](#_Toc236129904)

[Supplement Table 2: Age and sex distribution in the different populations.	3](#_Toc236129905)

[Supplement Table 3: Baseline characteristics of the ITT population - Allergy diagnostic /Total IgE, Eosinophils and sIgE.	4](#_Toc236129906)

[Supplement: Trial design - Inclusion and exclusion	5](#_Toc236129907)

[Supplement: Detailed description of secondary parameters	7](#_Toc236129908)

# Supplement Table 1: Overview of TIPP study populations.

Of the 100 enrolled patients, 86 were randomised to receive either tiotropium + ICS (n=44) or placebo + ICS (n=42) group; 14 patients were not randomised (Table 1 and CONSORT flowchart in figure 1). All randomised patients were included in the ITT and safety populations. The PP population was defined as a supportive analysis population and was not used for the primary efficacy analysis. The PP population comprised 31 patients (70% of the ITT) in the tiotropium group and 28 patients (67% of the ITT) in the placebo group. The most common protocol deviations were treatment compliance <80% (17%) and use of non-permitted medication (10%). Only one patient in the placebo group violated the inclusion criteria.

**Table 1: Overview of TIPP study populations.**

| **Population** | **Tiotropium + ICS (N=44)** | **Placebo +**  **ICS (N=42)** | **Not randomised (N=14)** | **Total (N=100)** |
| --- | --- | --- | --- | --- |
| **Enrolled Population** | 44 (100%) | 42 (100%) | 14 (100%) | 100 (100%) |
| Screening failure (no randomisation) | 0 (0%) | 0 (0%) | 7 (50%) | 7 (50%) |
| Trial completion at own request | 0 (0%) | 0 (0%) | 5 (36%) | 5 (36%) |
| Trial completion due to lost to follow-up | 0 (0%) | 0 (0%) | 1 (7%) | 1 (7%) |
| Trial completion due to other reason | 0 (0%) | 0 (0%) | 1 (7%) | 1 (7%) |
| **ITT Population** | 44 (100%) | 42 (100%) | 0 (0%) | 86 (86%) |
| Violation of inclusion/exclusion criteria | 0 (0%) | 1 (2%) | 0 (0%) | 1 (1%) |
|  |  |  |  |  |
| Meeting any exclusion criteria | 0 (0%) | 0 (0%) | 0 (0%) | 0 (0%) |
| Treatment not according to protocol | 1 (2%) | 1 (2%) | 0 (0%) | 2 (2%) |
| Compliance ≤80% | (18%) | 7 (17%) | 0 (0%) | 15 (17%) |
| Intake of not permitted medication as  defined in the protocol | 4 (9%) | 5 (12%) | 0 (0%) | 9 (10%) |
| Received wrong dose of treatment | 0 (0%) | 2 (5%) | 0 (0%) | 2 (2%) |
| Lack of efficacy | 1 (2%) | 0 (0%) | 0 (0%) | 1 (1%) |
| **PP Population** | 31 (70%) | 28 (67%) | 0 (0%) | 59 (59%) |
| **Safety Population** | 44 (100%) | 42 (100%) | 0 (0%) | 86 (86%) |
| *ICS* Inhaled corticosteroids; *ITT* Intention-to-treat; *N* Number of patients; *PP* Per-protocol.  Missing values are not included in calculation of percentages. | | | | |

All patients of the ITT population received treatment and attended the baseline, screening, and initial study visits (visit 2 and 3). A slight decline in visit attendance was observed over time, with 94% (N=80) of patients attending visit 6 and the lowest participation recorded at telephone contact (TC) (85% [N=73]). By the end of study, 95% (N=81) of patients attended the final visit, and 92% (N=79) completed the study regularly. A total of 8% (N=7) discontinued the study prematurely due to investigator decision (2% [N=2]), non-compliance (1% [N=1]), AEs (1% [N=1]), lost to follow-up (1% [N=1]) or other reasons (1% [N=1])).

# Supplement Table 2: Age and sex distribution in the different populations.

| **Population** | **Variable**  **(n=44)** | **Tiotropium + ICS**  **(N=44)** | **Placebo + ICS**  **(N=42)** | **Total**  **(N=86)** |
| --- | --- | --- | --- | --- |
| **ITT population** |  |  |  |  |
|  | Age [mean (SD)] | 3·2 (1·3) | 3·4 (1·3) | 3·3 (1·3) |
|  | Male (n, %) | 23 (52%) | 23 (55%) | 46 (53%) |
| **PP population** | n | 31 | 28 | 59 |
|  | Age [mean (SD)] | 3·1 (1·3) | 3·4 (1·4) | 3·3 (1·3) |
|  | Male (n, %) | 16 (52%) | 14 (50%) | 30 (51%) |
| **Safety population** | n | 44 | 42 | 86 |
|  | Age [mean (SD)] | 3·2 (1·3) | 3·4 (1·3) | 3·3 (1·3) |
|  | Male (n, %) | 23 (52%) | 23 (55%) | 46 (53%) |
| **Age group 1: 1–3 years** | n | 25 | 21 | 46 |
|  | Age [mean (SD)] | 2·2 (0·78) | 2·2 (0·70) | 2·2 (0·74) |
|  | Male (n, %) | 10 (40%) | 15 (71%) | 25 (54%) |
| **Age group 2: 4–5 years** | n | 19 | 21 | 40 |
|  | Age [mean (SD)] | 4·5 (0·51) | 4·6 (0·51) | 4·5 (0·51) |
|  | Male (n, %) | 13 (68%) | 8 (38%) | 21 (53%) |
| **Study centre: Frankfurt** | n | 18 | 17 | 35 |
|  | Age [mean (SD)] | 3·1 (1·21) | 3·5 (1·23) | 3·3(1·22) |
|  | Male (n, %) | 7 (39%) | 10 (59%) | 17 (49%) |
| **Centre: Other centres** | n | 26 | 25 | 51 |
|  | Age [mean (SD)] | 3·3 (1·4) | 3·4 (1·41) | 3·3 (1·39) |
|  | Male (n, %) | 16 (62%) | 13 (52%) | 29 (57%) |
| *ICS* Inhaled corticosteroids; *ITT* Intention-to-treat; *n* Number of patients; *PP* Per-protocol; *SD* Standard deviation. | | | | |

# Supplement Table 3: Baseline characteristics of the ITT population - Allergy diagnostic /Total IgE, Eosinophils and sIgE.

| **Variable** | **Value** | **Tiotropium + ICS (N=44)** | **Placebo + ICS (N=42)** | **Total (N=86)** |
| --- | --- | --- | --- | --- |
| **Total IgE kU/L** | Median (Q1–Q3) | 16·000 (16·00–57·00) | 16·000 (16·00–94·00) | 16·000 (16·00–94·00) |
| **Patients with IgE ≥100 (kU/L)** | N (%) | 9 (22%) | 9 (24%) | 18 (23%) |
| **Eosinophils 10^3^/µL** | Median (Q1–Q3) | 0·300 (0·12–0·37) | 0·250 (0·17–0·56) | 0·275 (0·15–0·41) |
| **Patients with EOS >300 µL** | N (%) | 11 (44%) | 12 (44%) | 23 (44%) |
| **Birch sIgE (KU/L)** | Median (Q1–Q3) | 0·10 (0·1–0·4) | 0·10 (0·1–0·4) | 0·10 (0·1–0·4) |
| **Patients with Birch sIgE ≥0**·**75 kU/L** | N (%) | 7 (18%) | 7 (20%) | 14 (19%) |
| **Grass sIgE (KU/L)** | Median (Q1–Q3) | 0·10 (0·1–0·4) | 0·10 (0·1–0·4) | 0·10 (0·1–0·4) |
| **Patients with Grass sIgE ≥0**·**75 kU/L** | N (%) | 5 (13%) | 4 (11%) | 9 (12%) |
| ***Dermatophagoides* *pteronyssinus* sIgE (KU/L)** | Median (Q1–Q3) | 0·10 (0·1–0·4) | 0·10 (0·1–0·4) | 0·10 (0·1–0·4) |
| **Patients with *Dermatophagoides pteronyssinus* sIgE ≥0**·**75 kU/L** | N (%) | 8 (20%) | 4 (11%) | 12 (16%) |
| **Cat sIgE (KU/L)** | Median (Q1–Q3) | 0·10 (0·1–0·4) | 0·10 (0·1–0·1) | 0·10 (0·1–0·2) |
| **Patients with Cat sIgE ≥0**·**75 kU/L** | N (%) | 6 (14%) | 3 (8%) | 9 (11%) |
| **Dog sIgE (KU/L)** | Median (Q1–Q3) | 0·10 (0·1–0·4) | 0·10 (0·1–0·2) | 0·10 (0·1–0·4) |
| **Patients with Dog sIgE ≥0**·**75 kU/L** | N (%) | 3 (7%) | 2 (5%) | 5 (6%) |
| ***Cladosporium* sIgE (KU/L)** | Median (Q1–Q3) | 0·10 (0·1–0·1) | 0·10 (0·1–0·1) | 0·10 (0·1–0·1) |
| **Patients with *Cladosporium* sIgE ≥0**·**75 kU/L** | N (%) | 0 (0%) | 0 (0%) | 0 (0%) |
| ***Alternaria* sIgE (KU/L)** | Median (Q1–Q3) | 0·10 (0·1–0·1) | 0·10 (0·1–0·1) | 0·10 (0·1–0·1) |
| **Patients with *Alternaria* sIgE ≥0**·**75 kU/L** | N (%) | 2 (5%) | 1 (3%) | 3 (4%) |
| **Cow`s milk sIgE (KU/L)** | Median (Q1–Q3) | 0·10 (0·1–0·3) | 0·10 (0·1–0·4) | 0·10 (0·1–0·4) |
| **Patients with**  **Cow´s milk sIgE ≥0**·**75 kU/L** | N (%) | 3 (7%) | 2 (6%) | 5 (7%) |
| **Hen`s egg sIgE (KU/L)** | Median (Q1–Q3) | 0·10 (0·1–0·3) | 0·10 (0·1–0·4) | 0·10 (0·1–0·3) |
| **Patients with**  **Hen´s egg sIgE ≥0**·**75 kU/L** | N (%) | 4 (10%) | 3 (9%) | 7 (9%) |
| **Peanut sIgE (KU/L)** | Median (Q1–Q3) | 0·10 (0·1–0·2) | 0·10 (0·1–0·4) | 0·10 (0·1–0·3) |
| **Patients with Peanut sIgE ≥0**·**75 kU/L** | N (%) | 3 (8%) | 4 (11%) | 7 (9%) |
| *ICS* Inhaled corticosteroids; *ITT* Intention-to-treat; *N* Number of patients; *sIgE* specific immunoglobulin E; *Q* Quartile  Missing values are not included in calculation of percentages.  Pollen allergens all: Grass, birch, *Alternaria* and *Cladosporium* pollen  Perennial allergens all: mites, cat and dog  Food allergens all: egg, milk and peanut  Analysis Set: ITT (N=86) | | | | |

# Supplement: Trial design - Inclusion and exclusion

| **Treatment/intervention** | In general, Tiotropium is indicated as a maintenance bronchodilator treatment to relieve symptoms of patients with chronic obstructive pulmonary disease. Spiriva Respimat^®^ is indicated as add-on maintenance bronchodilator treatment in patients aged 6 years and older with severe asthma who experienced one or more severe asthma exacerbations in the preceding year.  In the present study, the lower dose of 2·5 µg tiotropium was used to be effective and to save cost for better patient care. After signing informed consent, eligible patients were randomised at V2 to either the control group (placebo) or the intervention group (Tiotropium) according to the randomisation list prepared by IZKS Mainz. Patients took two puffs of the medication (Placebo or tiotropium) daily, corresponding to a total daily dose of 2·5 µg placebo/tiotropium for a 52-week treatment period.  Each inhaler contained 60 puffs. The trial medication was labelled according to §5 GCP regulation—including an individual medication number—by Almac Ltd., Craigavon. A sufficient number of inhalers were packed into boxes by the pharmacy according to the randomisation list. After randomisation, parents were given a medication box, corresponding to their randomisation number, packed with inhalers according to their treatment group. To ensure that there was sufficient medication between visits, care was taken to provide parents with a sufficient number of inhalers.  The investigator took inventory and acknowledged the receipt of all shipments of the trial medication.  All trial medication was kept in a locked area with access restricted to designated trial staff. The trial medication was stored dry and according to manufacturer’s instructions at room temperature in its closed primary packaging separated from trading goods. The investigator also kept accurate records of the quantities of trial medication dispensed, used, and returned by each patient on the drug accountability form. The site monitors periodically checked the supplies of trial medication held by the investigator to verify the correct accountability of all trial medication used. At the end of the trial, all unused trial medication and all medication containers were destroyed at the participating site. Destruction of medication was recorded in the corresponding log. Expired medication during the study was destroyed by the centres and the pharmacy at the University Medical centre Mainz, if applicable. A destruction protocol was prepared by the centres and pharmacy, respectively, and provided to the sponsor, IZKS Mainz and Boehringer Ingelheim. If patients were still on treatment at this time, these patients received a corresponding number of new inhalers—according to their treatment allocation—with a new expiration date. Destruction of study medication was arranged first after permission by the sponsor. The investigator ensured that a final drug accountability report (Centre Drug Accountability) was prepared. |
| --- | --- |
| **Terms of comparison/ Comparator** | **Comparative product (placebo)** Formulation: \| Placebo solution (benzalkonium chloride, disodium edetate, purified water, hydrochloric acid 3·6%) for inhalation; Manufacturer: \| Boehringer Ingelheim Pharma GmbH & Co.KG |
| **Total number of patients** | - Calculated sample size = 152 patients, after protocol 4.0 was amended - Enrolled = 100 patients - Randomised = 86 patients - Drop-outs = 7 patients |
| **Study population** | All patients whose parents/legal guardians signed informed consent were considered as enrolled. All randomised patients were included in the intention-to-treat (ITT) population. This population was the primary analysis population. Within the ITT population analyses patients were assigned to the treatment to which they had been randomised.  To be eligible for the per-protocol (PP) population, patients had to fulfil at least the following criteria:   - No major protocol violations, which were - Violation of inclusion or exclusion criteria - Patient did not receive the treatment she/he was assigned to by randomisation - Intake of not permitted medication as defined in the protocol - No concomitant treatment with oral antibiotics longer than 14 consecutive days or any monoclonal antibody at any time. - Treatment compliance of at least 80% referring to the visits that were attended.   The safety population comprised all patients who received at least one dose of trial treatment. In analyses of the safety population, patients were assigned to the treatment which they actually received. The analysis populations were defined before unblinding the study.  Special cases:   - Patient 02/01 received the wrong dose by mistake (parents’ fault) and prematurely discontinued study medication. This patient was excluded from the PP population. - Patient 11/01 received a dose that was too low and therefore was excluded from the PP population. - Patient 01/15 accidentally received and used one inhaler with randomisation number 015 instead of number 010 (after the patient who actually received inhaler 015 had already completed the study). Unblinding revealed that inhaler 015 did not belong to the same study arm as 010. Therefore, the patient was excluded from the PP population. - Patient 01/43 accidentally received tiotropium in addition to the study treatment and therefore was excluded from the PP population. |
| **Inclusion criteria** | Patients who met all of the following criteria were considered for admission to the trial:   1. All patients' parents (or legal guardians) must have had signed and dated an informed consent, consistent with ICH-GCP guidelines and local legislation prior to participation in the trial. In addition, an appropriate assessment in accordance with local regulations or guidelines or both before enrolment in the study and prior to the beginning of any study-specific procedures had to be obtained from the child. 2. Ability of parents/legal guardians to understand nature, importance and individual consequences of the clinical trial. 3. Male or female patients with preschool asthma aged between 1–5 years   (<6 at visit 1).   4. Physicians diagnosed asthma of at least 6 months’ history of asthma symptoms, including (but not limited to) wheezing, cough, and/or shortness of breath. 5. All patients must have been on maintenance treatment with an ICS at a stable dose, either as mono treatment or in combination with another controller medication, for at least 4 weeks before visit 1. 6. Patient was hospitalised due to acute severe asthma and/or was treated with at least two courses of systemic steroids (three days of oral steroids or one day of rectal prednisolone 100mg (Rectodelt^®^)) in the last 24 months before visit 1. 7. All patients must have been symptomatic (partly or uncontrolled) as defined by the GINA guideline for children aged 5 years and younger in the four weeks prior to screening (visit 1) and randomisation  (visit 2) despite treatment with ICS. 8. Patients must have been able to inhale from the Respimat^®^ inhaler (with a spacer). |
| **Exclusion criteria** | Patients who presented with any of the following criteria were not included in the trial:   1. Patients with a significant disease other than asthma such as, but not limited to, the following diagnoses: cystic fibrosis, bronchopulmonary dysplasia, primary immune deficiency, congenital heart disease, parasitic disease, and foreign body aspiration. 2. Patients with clinically relevant abnormal screening haematology or blood chemistry were excluded if the abnormality defined a significant disease as defined in the exclusion criterion. 3. Patients with a history of congenital or acquired heart disease, or patients who have been hospitalised for cardiac syncope or failure during the past year. 4. Medical or psychological conditions that would have jeopardised adequate and orderly completion of the trial. 5. Patients with known hypersensitivity to anticholinergic drugs, orany othercomponents of the tiotropium inhalation solution. 6. Patients with severe acute asthma exacerbation or severe respiratory tract infection defined as systemic steroid intake or hospitalisation in the four weeks before visit 1 (screening). 7. Patients who have previously been randomised in this study  or are currently participating in another study. 8. Patients with moderate to severe renal impairment as tiotropium is a predominantly renally excreted drug. 9. Patients with chronic systemic steroids use, and patients treated with monoclonal antibodies like Omalizumab and Dupilumab. |

# Supplement: Detailed description of secondary parameters

**Caregiver Global Impression of Change (CGI-C)**

CGI-C ratings were comparable between the two treatment groups. Substantial improvement was reported in approximately half of the patients in each group (50% [N=21] in the tiotropium + ICS group vs. 51% [N=20] in the placebo + ICS group). Moderate improvement was reported more often in the tiotropium + ICS group (31% [N=18] vs. 21% [N=8]), while minimal improvement or no change was observed in 22% (N=18) of patients overall. Worsening was rare in both groups, with only one case of moderate worsening reported in the placebo + ICS group. Overall, caregiver-reported global impression ratings were similar between groups.

**Potential markers for treatment response** (in patients aged 4-5 years)

The exploratory biomarker analysis included markers of type 2 inflammation (blood eosinophils and total IgE) and a broad panel of allergen-specific IgE sensitisation markers (including *Alternaria*, birch, cat, *Cladosporium*, cow’s milk, *Dermatophagoides pteronyssinus*, dog, grass, hen’s egg, and peanut). No consistent or clinically meaningful treatment-related differences were observed across biomarkers. As biomarker measurements were restricted to the subgroup of children aged 4–5 years and were not available for all participants within this subgroup, several biomarkers had substantial proportions of missing data.

**Subgroup analyses**

For subgroup analyses, the primary analysis was repeated with additional adjustment for the respective subgroup and inclusion of an interaction term between treatment and the subgroup. These subgroup and interaction analyses were exploratory and should be interpreted with caution given the limited sample size within subgroups and the resulting reduced statistical power.

**a) Time to first severe exacerbation by sex**

For male patients, the HR for tiotropium + ICS vs. placebo + ICS was 1·67 (95% CI: 0·63–4·43), and for female patients 0·49 (95% CI: 0·18–1·34). The interaction between treatment and sex was not statistically significant (p=0·095). In the placebo + ICS group, male patients had a significantly lower risk of exacerbation compared to female patients (HR: 0·33, 95% CI: 0·11–0.94; p=0·038). The covariates age group and centre group did not show any significant effects.

**b) Time to first severe exacerbation by allergic status**

Among allergic patients, four severe exacerbations occurred in the placebo + ICS group and seven in the tiotropium + ICS group. Among non-allergic patients, 11 events occurred in the placebo + ICS group and ten in the tiotropium + ICS group. The HR for tiotropium + ICS versus placebo + ICS was 1·41 (95% CI: 0·40–4·97) in allergic patients and 0·69 (95% CI: 0·29–1·65) in non-allergic patients. The interaction between treatment and allergic status was not statistically significant (p=0·37). When comparing allergic vs. non-allergic patients within treatment groups, no statistically significant differences in risk were observed (tiotropium + ICS, HR: 2·59, 95% CI: 0·75–8·95; p=0·71). The covariates age group and centre group did not show any significant effects.
